# The Many Ages of Microbiome–Gut–Brain Axis

**DOI:** 10.3390/nu14142937

**Published:** 2022-07-18

**Authors:** Daniela Ratto, Elisa Roda, Marcello Romeo, Maria Teresa Venuti, Anthea Desiderio, Giuseppe Lupo, Enrica Capelli, Anna Sandionigi, Paola Rossi

**Affiliations:** 1 Department of Biology and Biotechnology “L. Spallanzani”, University of Pavia, 27100 Pavia, Italy; daniela.ratto@unipv.it (D.R.); drmarcelloromeo@gmail.com (M.R.); mariateresa.venuti01@universitadipavia.it (M.T.V.); 2 Laboratory of Clinical & Experimental Toxicology, Pavia Poison Centre, National Toxicology Information Centre, Toxicology Unit, Istituti Clinici Scientifici Maugeri IRCCS, 27100 Pavia, Italy; elisa.roda@unipv.it; 3 Department of Earth and Environmental Sciences, University of Pavia, 27100 Pavia, Italy; anthea.desiderio01@universitadipavia.it (A.D.); giuseppe.lupo01@universitadipavia.it (G.L.); enrica.capelli@unipv.it (E.C.); 4 Department of Biotechnology and Biosciences, University of Milano-Bicocca, 20126 Milan, Italy; anna.sandionigi@quantiaconsulting.com; 5 Quantia Consulting S.r.l., Via Petrarca 20, 22066 Mariano Comense, Italy

**Keywords:** gut microbiome, aging, frailty, cognitive decline, inflammaging, eubiosis, dysbiosis, adaptive mechanism, maladaptive mechanism

## Abstract

Frailty during aging is an increasing problem associated with locomotor and cognitive decline, implicated in poor quality of life and adverse health consequences. Considering the microbiome–gut–brain axis, we investigated, in a longitudinal study, whether and how physiological aging affects gut microbiome composition in wild-type male mice, and if and how cognitive frailty is related to gut microbiome composition. To assess these points, we monitored mice during aging at five selected experimental time points, from adulthood to senescence. At all selected experimental times, we monitored cognitive performance using novel object recognition and emergence tests and measured the corresponding Cognitive Frailty Index. Parallelly, murine fecal samples were collected and analyzed to determine the respective alpha and beta diversities, as well as the relative abundance of different bacterial taxa. We demonstrated that physiological aging significantly affected the overall gut microbiome composition, as well as the relative abundance of specific bacterial taxa, including *Deferribacterota*, *Akkermansia*, *Muribaculaceae*, *Alistipes*, and *Clostridia VadinBB60*. We also revealed that 218 amplicon sequence variants were significantly associated to the Cognitive Frailty Index. We speculated that some of them may guide the microbiome toward maladaptive and dysbiotic conditions, while others may compensate with changes toward adaptive and eubiotic conditions.

## 1. Introduction

Aging is a natural process affecting all living organisms, and it is characterized by a deterioration in physiological processes, leading to a higher probability to develop several disorders, i.e., cancer, metabolic, cardiovascular, and neurodegenerative diseases [1,2]. Indeed, aging is characterized by a decline in cognitive and locomotor functions [3]. Among different cognitive performances, the recognition memory has been described as a major component of mammalian and human personality [4,5,6], and it is early and heavily affected during aging.

In the elderly, frailty is defined as a clinical state with an increased vulnerability to stressors, thus exposing the organism to negative health-related outcomes, in the absence of recognized pathologies [7]. Frailty is a multisystem dysregulation leading to decreased physiological reserve [8,9]. Thus, frailty is related to aging, but it does not reflect chronologic age, showing big heterogeneity among subjects [10]. The age-related frailty is principally associated with phenotypic and/or locomotor impairment, both in humans and in animal models [8,11]. Currently, however, more and more attention is paid to the cognitive impairment that occurs during aging, which temporally follows the decline in physical functions in wild-type mice [3]. The development of a frailty score is crucial and necessary to monitor the decline in physiological functions during aging [11].

Further investigations are mandatory to develop strategies to prevent and/or reverse frailty, cognitive impairment, and disability in the elderly [12].

The main cause of aging is usually considered the time-dependent increase in cellular damages [1]. Among others, the most considered biomarkers of aging in mammalians are DNA damage accumulation, apoptosis resistance, genomic instability, telomere shortening, epigenetic alterations, deregulated nutrient sensing, mitochondrial dysfunction, stem cell exhaustion, and altered intercellular communication [1,13,14]. The hallmarks of aging lead to both tissue function decrease and inflammation, in particular in the gastrointestinal system, increasing the predisposition to gut-associated diseases by causing alterations in the gut microbiome composition in elderly people [15]. The aging-related changes in gut physiology contribute to microbiome change, with the disappearance, the persistence, or the outgrowth of several specific microbes. In particular, the aging microbiota appears to be suffering from reduced resilience that can be problematic, but it can also provide an opportunity [16]. Furthermore, changes in microbiota composition during aging could be due to both a maladaptive and dysbiotic or an adaptive and eubiotic condition of the gut microbiome in a delicate balance between inflammaging, immunosenescence, and ecological homeostasis over time. Specifically, an association between aging and frailty and a change in *Akkermansiaceae, Lachnospiraceae*, *Rikenellaceae*, and *Ruminococcaceae,* among others, has been described [16].

In particular, inflammaging, a low-grade chronic inflammation during aging, was recently considered a trigger of the leaky gut and of the gut microbiome dysbiosis which characterize the elderly. According to this, the relative abundance of several microbes in the gut is dependent on cytokine and chemokine levels [16].

In physiological conditions, the human gut is inhabited by mutualistic bacterial, fungal, archaeal, viral, and protozoal communities, which together form the gut microbiome [17,18]. A healthy gut microbiome that dynamically interacts to host contains two predominant phyla, *Bacteroidetes* and *Firmicutes*, followed by the *Actinobacteria*, *Proteobacteria*, and *Verrucomicrobia* phyla [19]. Even though the general gut microbial composition remains constant, the gut microbiota exhibits temporal and spatial differences in microorganism distribution during the lifespan [20]. Indeed, several factors, such as host diet, lifestyle, environmental exposure, genotype, and physio-pathological status influence gut microbiome composition which changes dynamically throughout the entire life of a host [21].

This symbiotic and mutualistic superorganism, composed by the microbiome and host, has important effects on host health [20,22,23]. Indeed, increasing interest is focused toward the gut microbiota: the dysbiosis has been associated with several human diseases, such as inflammatory bowel disease (IBD), irritable bowel syndrome (IBS), obesity, diabetes, allergic disorders, and neurodegenerative diseases [18,24,25]. Therefore, intervention on the gut microbiota composition is fundamental to prevent and treat different diseases, including cognitive frailty during aging. Impairment of the microbiota–gut–brain axis has been associated with several disorders, including neuropsychiatric diseases. Hence, mounting evidence supports the hypothesis that gut microbiome dysbiosis is implicated in the onset of cognitive impairment and frailty [10,24,26]. Furthermore, recent studies proposed inflammaging as a critical component in the onset and progression of frailty and demonstrated that the gut microbiota becomes pro-inflammatory over time during aging [10].

Herein, we investigated the intersection of age, the gut microbiome, and cognitive frailty with a longitudinal study in a mouse model of physiological aging. First, we addressed the differences in the gut microbiome in alpha and beta diversities during physiological aging, also describing the changes in the relative abundance of specific bacterial taxa. In parallel, we monitored the Cognitive Frailty Index during aging by using spontaneous behavioral tests for evaluating recognition memory. Finally, we recognized the specific changes in microbiome composition due to the cognitive frailty, and we tried to describe them from a functional point of view.

## 2. Materials and Methods

### 2.1. Mice and Behavioral Tests

Fourteen wild-type male mice (C57BL-6J) were obtained from Charles River, Italy. The mice were acclimated to their environmental conditions for one month before starting experiments, which were conducted in the Animal Care Facility at the University of Pavia. The mice were individually housed in plastic cages with an automatically controlled light-dark cycle: the dark period lasted from 07:00 to 19:00 and the light period was from 19:00 to 07:00. Water and food were furnished *ad libitum*. All experimental protocols and animal handling were carried out in strict conformity with European Council Directive 2010/63/EU and with the guidelines set by the Ethics Committee of Pavia University (Ministry of Health, License number 774/2016-PR).

The murine cognitive performance was studied accessing the knowledge component of recognition memory [6], tested through two spontaneous behavioral tests which consisted of (i) recognizing a new object (namely, novel object recognition (NOR) test) in an open arena or (ii) in exploring a new environment (namely, emergence test) in non-stressful conditions. Emergence and NOR tasks were carried out in accordance with Brandalise et al., 2017 [27]. Concerning the emergence test, the analyzed parameters were latency for the first exit(s), number of exits, and time of exploration(s). Regarding the NOR task, the parameters of interest were the discrimination capabilities (discrimination index, DI) for number and time of approaches. For all selected parameters, we obtained the corresponding Cognitive Frailty Index (FI), then the Cognitive FI related to the test (emergence or NOR), and finally the overall Cognitive FI, as previously reported [3].

**Figure 1:**
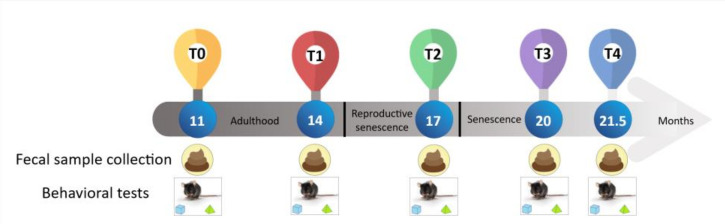
Flow diagram of experimental plan with the chosen time points: 11, 14, 17, 20, and 21.5 months of mice age, corresponding to T0, T1, T2, T3, and T4, respectively. It has to be noted that T0 and T1 belonged to adulthood, T2 to reproductive senescence, and T3 and T4 to senescence. At each time point, behavioral tests were performed and stool samples were collected).

In detail, spontaneous behavioral tests in mice were performed at five experimental time points: T0 = 11, T1 = 14, T2 = 17, T3 = 20, and T4 = 21.5 months of age. Notably, T0 and T1 belonged to adulthood, T2 to reproductive senescence, while T3 and T4 occurred during senescence. At the same experimental times, animals were weighed, and the fecal stool samples were collected and stored at −80 °C. The experimental design is summarized here below in Figure 1.

### 2.2. Bacterial DNA Extraction and 16s rRNA Sequencing

Total microbiota genomic DNA in the mice stools was extracted by using a QIAamp DNA stool mini kit (Qiagen, Dusseldorf, Germany) in accordance with the manufacturer’s instructions. DNA quantification was performed by using Qubit FluorometerTM (Invitrogen, Molecular Probes, USA). Libraries for 16S rDNA amplicons sequencing of the V3 and V4 regions were obtained by using PCR primers containing a barcode (V3 Forward: 5′-CCTACGGGNGGCWGCAG-3′; V4 Reverse: 5′-GACTACHVGGGTATCTAATCC-3′). Furthermore, for preparing the amplicons for the sequencing by MiSeq Illumina, we used the specific forward (V3 F: 5′-TCGTCGGCAGCGTCAGATGTGTATAAGAGACAG-3’) and reverse (V4 R: 5’-GTCTCGTGGGCTCGGAGATGTGTATAAGAGACAG-3’) adapters. Finally, we performed the PCR analysis by using Bio-Rad MJ Mini Personal Thermal Cycle, and PCR amplicons were sequenced by MiSeq Illumina, relying on the BMR Genomics SRL of Padova.

### 2.3. Illumina Data Processing and Microbiota Characterization

The raw paired-end FASTQ reads were imported into the Quantitative Insights Into Microbial Ecology 2 program (QIIME2, ver. 2020.2.01) [28] and demultiplexed using the native plugin. The Divisive Amplicon Denoising Algorithm 2 (DADA2) [29] was used to quality filter, denoise, and mergepair the data and remove chimeric sequences.

The resulting amplicon sequence variants (ASVs) with less than a 50× coverage were discarded from further analyses. The classification of the obtained ASVs was run using the feature-classifier plugin [30], implemented in QIIME2 against the SILVA SSU non-redundant database (138 release) [31], adopting a consensus confidence threshold of 0.7.

### 2.4. Data Analysis and Statistics

All data are expressed as mean ± SEM.

The analysis on the bacterial diversity, and the corresponding figures, was performed using the phyloseq R package [32]. Microbiota diversity was described in terms of within (alpha) and between (beta) sample diversities. The Shannon diversity index (SDI) and Faith’s phylogenetic distance (PD) [33] alpha diversity metrics were calculated to estimate the variation of bacterial diversity at the different time points. Values were compared using the pairwise Wilcoxon rank-sum test (WRST) [34] was used pairwise to determine whether the value of a specific metric (Shannon index and Faith’s PD) changed significantly between different time points.

Beta diversity was estimated with quantitative distance metrics using the diversity function in the phyloseq R package. We estimated the Bray-Curtis dissimilarity indices by sampling 10,000 reads per sample [32] based on estimated rarefaction curves (see Supplementary Figure S1).

Variance partitioning and significant Cognitive FI variation over time were determined by performing linear mixed effects (LME) models with the lme4 R package [34].

Variance partitioning and significance of bacterial communities’ diversity variation over time and with respect to the Cognitive FI were determined by performing PERMANOVA test using the Adonis function implemented in the vegan R package. To overcome repeated measures, the variable that describes the mice was defined as strata.

Canonical analysis of principal coordinates (CAP) was computed using the capscale function from the vegan R Package [35,36]. To highlight the relationship between changes in community tolerance and shifts in community composition, a constrained ordination was performed by distance-based redundancy analysis with time, with Cognitive FI as the constraint variable. In addition, we used the unconstrained ordination method (principal coordinate analysis) to visualize patterns in bacterial community compositions. The significance of constraint variables was tested with a permutation test (number of permutations = 10,000) using the ANOVA function implemented in the vegan R package.

The structure of microbial communities was explored by the non-multidimensional scaling (NMDS) ordination approach [37].

The DESeq2 R package [38] was used to identify the bacteria with the most significant changes in ASV differential abundance considering the Cognitive FI. Improvements to the stability and dispersion of the counts (variance) were required before it was possible to calculate the differential abundances for the different species present in the samples being compared. To this end, we used the estimated size factors function in DESeq2 to transform the stabilization of the variance. The differential abundances were measured with the log2foldchange value, and the different conditions were compared using the Wald test with the Benjamini–Hochberg correction (Q parameter = 0.1, FDR < 10%). The differential abundance measurements were statistically significant if the adjusted *p*-value was <0.05.

The metacoder R package was used to generate tree plots [39].

All the graphical plots were generated by the ggplot2 R package [40]. The method for calculating ellipses is implemented in the ggplot2 package in the stat ellipse function based on John Fox and Sanford Weisberg (2011) [41].

The sequences generated in this study were deposited into the EMBL-EBI database in the PRJEB54046 project.

## 3. Results

### 3.1. Sequencing Data Results

We performed a longitudinal study on fourteen mice sampled five times (from T0 to time T4). The gut microbiome of those 70 samples was examined by sequencing of the bacterial 16S rRNA gene. After quality control analysis, sequences of 70 libraries resulted in 1,748,748 million sequence reads, ranging from 564 to 43,360 reads, with a median of 25,801 for a sample (see Supplementary Figure S2). No reads were reported in negative controls. A total of 1458 ASVs were identified.

### 3.2. Aging Affects Overall Gut Microbiome Composition

**Figure 2:**
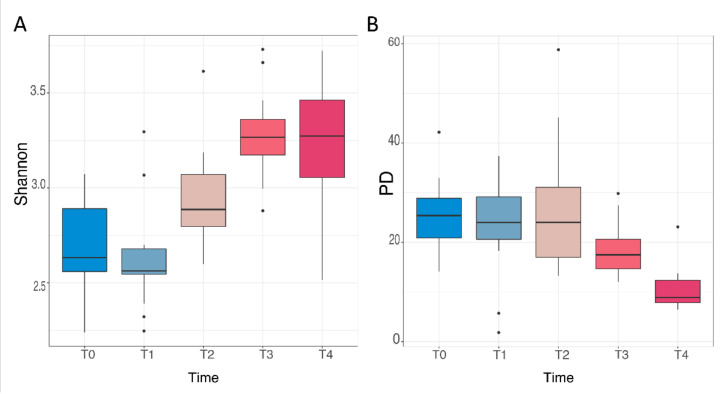
Alpha diversity distribution box plots. In (**A**): Shannon diversity index (SDI) estimated for each time point. In (**B**): Faith’s phylogenetic distance (PD) estimated for each time point. (Number of mice: T1 *n* = 14, T2 *n* = 12, T3 *n* =14, and T4 *n* =13).

Stool profiling revealed large microbiome composition differences over time during the mice lifespans. The alpha diversity (microbial diversity within the sample) changed significantly during physiological aging, but Shannon diversity index (SDI) and Faith’s phylogenetic distance (PD) exhibited opposite behavior (Figure 2).

There was a significant increase in terms of biodiversity observed with Shannon index between time T1 vs. T2 (Wilcoxon rank-sum test (WSRT), *p* = 0.007) and between T2 vs. T3 (WSRT, *p* = 0.011); on the contrary, any significant variation was determined between T0 and T1, as well as between T3 and T4 (Table 1 and Table S1). Supplementary Figure S3 reports the Simpson index variation during aging, which showed the same trend as the Shannon index.

Concerning Faith’s PD, which considers phylogenetic information, we observed a decreasing trend, even though a significant variation was perceived only between T3 and T4 (WSRT, *p* = 0.0002). As regards the other time points, any significant change was measured (Table 1 and Table S1).

**Figure 3:**
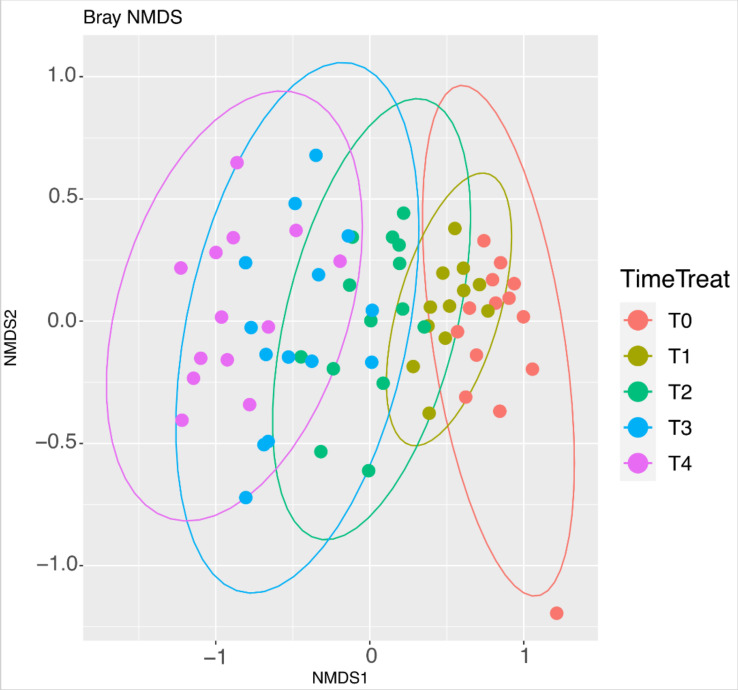
Non-metric multidimensional scaling (NMDS) at different time points (T0–T1–T2–T3–T4). Colors in the bidimensional NMDS plot are used according to the different sample origin as shown in the legend. The ordinate analysis is based on the Bray-Curtis distance matrix. The graphical plot and the ellipses were generated by ggplot2 R package implemented with stat ellipse function. (Number of animals: T1 *n* = 14, T2 *n* = 12, T3 *n* =14, and T4 *n* =13). Colors in the graphs are reported according to the different experimental times as shown in the figure labels.

To obtain a complete picture of the variation over time of the measured diversity, we estimated the magnitude of change among successive time points by considering the beta diversity, a measure of similarity or dissimilarity of different microbiome communities. As shown in Figure 3 (NMDS based on Bray-Curtis distance matrix; stress value = 0.13), the murine microbiomes were similar at each experimental time but changed over the mice lifespan. This fact determined the identification of five consequential clusters specific for each time (Figure 3). This longitudinal effect of beta diversity modification increased further and further over time, creating a large gap between the T0 and the T4 group (see results in Table 2 and Table S2).

### 3.3. Detailed Microbiome Composition and Aging Distribution

A total of 1458 ASVs were identified throughout the whole experimental time. Of these, 76% was assigned at least at the phylum level, and specifically, we found 13 phyla, 20 classes, 54 orders, 76, families, and 145 genera.

**Figure 4:**
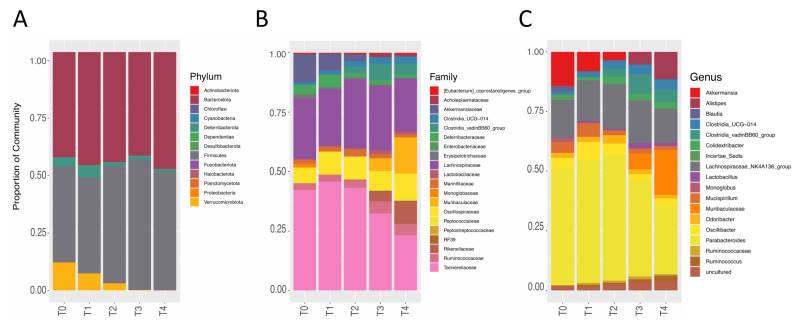
Bar chart regarding the distribution of the most abundant phyla (**A**), families (**B**), and genera (**C**). The proportion of stack in bar chart corresponds to the total amount of reads of the most abundant phyla, families and genera. (Number of animals: T1 *n* = 14, T2 *n* = 12, T3 *n* = 14, and T4 *n* = 13).

We recorded a continuum in the phyla taxonomic level during aging. During the entire aging process, the most represented phylum was *Bacteroidota* followed by *Firmicutes* (Figure 4A). The greatest changes during aging were the disappearance of the *Verrucomicrobiota* phylum from 13.84% at T0 to less than 0.01% at T4 and the decrease in *Deferribacterota* from 4.9% at T1 to 1.2% at T4.

At the family and genus taxonomic levels, the greatest variations in microbiome composition were observed during aging (Figure 4B,C). The most represented families during the entire physiological aging process were *Tannerellaceae* and *Lachnospiraceae*, of *Bacteroidota* and *Firmicutes* phyla, respectively. Some families underwent a strong variation during physiological aging. In particular, the *Akkermansiaceae* (*Verrucomicrobiota* phylum) decreased from 13.86% at T0 to 3.7% at T2 and almost disappeared at T3 and T4. On the contrary, the *Muribaculaceae* (*Bacteroidota* phylum) appeared at T3 at a percentage of 5.3% and increased even more to 14.4% at T4.

**Figure 5:**
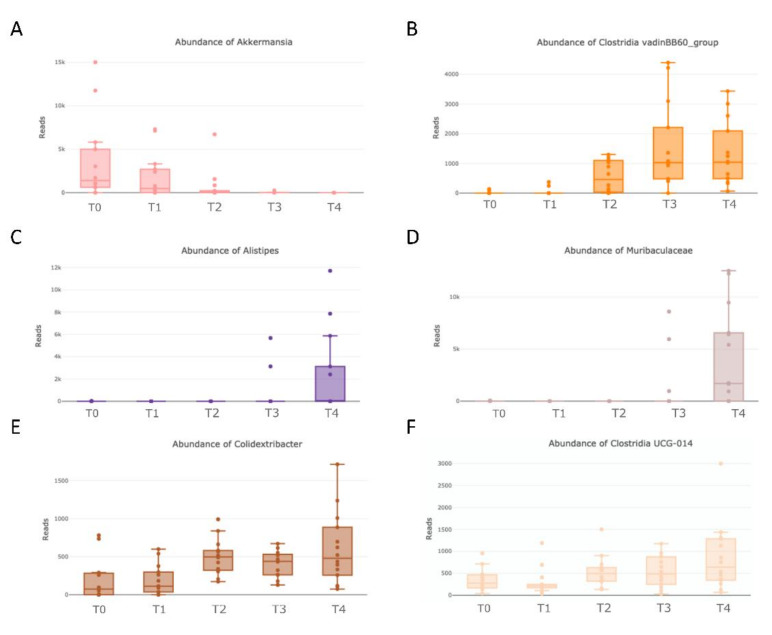
Box plots representing the relative abundance of genera (**A**) *Akkermansia*, (**B**) *Clostridia_vadinBB60_group*, (**C**) *Alistipes*, (**D**) *Muribaculaceae,* (**E**) *Colidextribacter*, and (**F**) *Clostridia UCG-014*. (Number of mice: T1 *n* = 14, T2 *n* = 12, T3 *n* =14, and T4 *n* =13).

The most represented genera during the mice lifespan were *Parabacteroides* (*Bacteroidota* phylum; *Tannerellaceae* family) and *Lachnospiraceae_NK4A136_group* (*Firmicutes* phylum; *Lachnospiraceae* family) which did not undergo statistical fluctuations (Figure 4C). The genera *Akkermansia* (*Verrucomicrobiota* phylum; *Akkermansiaceae* family) disappeared between adulthood and senescence (Figure 4C and Figure 5A, respectively), mirroring the variation at the phylum and family level. During senescence (at T3), we witnessed a statistically significant increase in the genus *Clostridia_vandinBB60* (*Firmicutes* phylum; *Clostridia* family) (from 0.043% at T0 to 4.2% at T4) (Figure 4C and Figure 5B, respectively), paralleled by a similar sized increase of the genus *Alistipes* (from 4.6% at T0 to 9.8% at T4) (Figure 4C and Figure 5C, respectively), representing the entire *Rikenellaceae* family (*Bacteroidota* phylum). Furthermore, *Muribaculaceae* genera (*Bacteroidota* phylum, *Muribaculaceae* family) appeared in senescence (at T3 5.17% and at T4 13.57%, Figure 4C and Figure 5D, respectively). Finally, we perceived a slight and continuing non-significant trend of growth during aging for *Colidextribacter* (*Firmicutes* phylum) and *Clostridia UCG-014* genera (*Firmicutes* phylum, *Clostridia* family) (Figure 4C and Figure 5E,F, respectively). 

**Figure 6:**
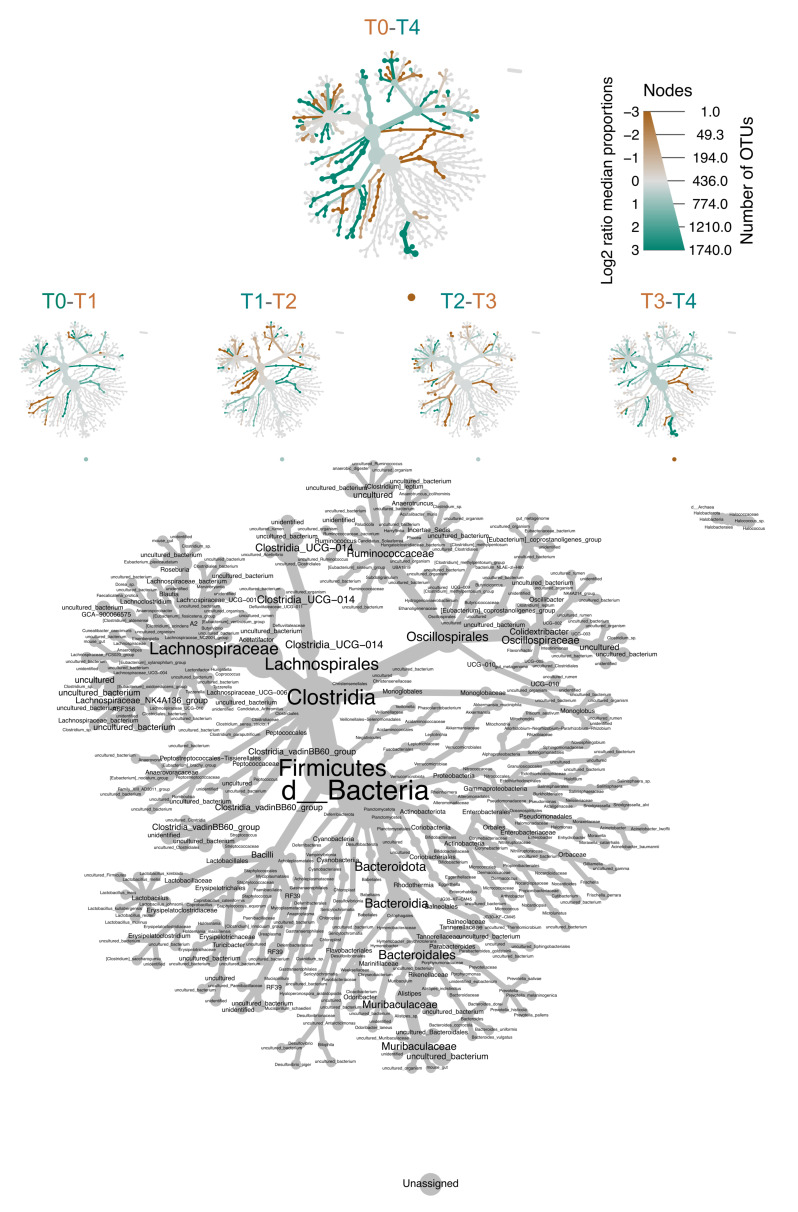
A differential heat tree based on the Wilcoxon rank-sum test, indicating which taxa were more abundant in each experimental time. Phyla, classes, orders, families, and genera are represented. Node label is the taxon name, node size is the number of ASVs, and node color is the abundances of the indicated phylum, class, order, family, or genus. A taxon colored brown was more abundant in the time points colored in brown and a taxon colored in green was more abundant in the time points colored in green, as reported in the legend. The tree differential plots were generated using the metacoder R package. (Number of animals: T1 *n* = 14, T2 *n* = 12, T3 *n* =14, and T4 *n* =13).

To investigate in more detail the community structure, we displayed our data as a heat tree map, where it is possible to follow all the taxonomic levels from phyla to species. Furthermore, in the heat tree map, the proportions were subjected to statistical testing. Based on the Wilcoxon rank-sum test, Figure 6 shows the heat tree map describing the different taxa at each experimental time during aging. The size and color of nodes and edges correlate with the abundance of bacteria at each experimental time. The heat tree map describes both the relative changes in sequential time and the whole change between adulthood (T0) and senescence (T4) (Figure 6).

### 3.4. Aging and Cognitive Frailty Index on Microbiome Composition

**Figure 7:**
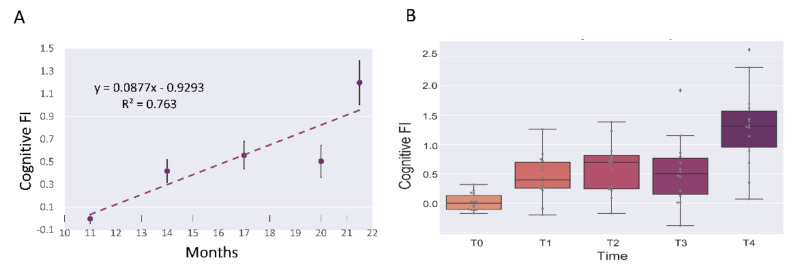
Mean cognitive decline, reported as Cognitive Frailty Index (FI), during physiological aging (*n* = 14). (**A**): linear least-squares regression analysis of Cognitive FI during mice lifespan. (**B**): median value of Cognitive FI during mice lifespan.

We investigated the knowledge component of the recognition memory, which significantly worsened during aging, as previously reported [3]. In particular, by using specific spontaneous behavioral tests (emergence and NOR tasks), we evaluated the mice’s capabilities in distinguishing an environment (emergence task) or an object (NOR task) as familiar or novel, during the aging process. Altogether, the overall Cognitive FI (used for evaluating the cognitive impairment) values significantly increased during physiological aging in wild-type mice (Figure 7), indicating that physiological aging was accompanied by cognitive decline. Remarkably, there was a significant increasing effect of time on Cognitive FI (See Supplementary Table S3 for LME model details), confirming the direct correlation between the Cognitive FI and the aging process.

**Figure 8:**
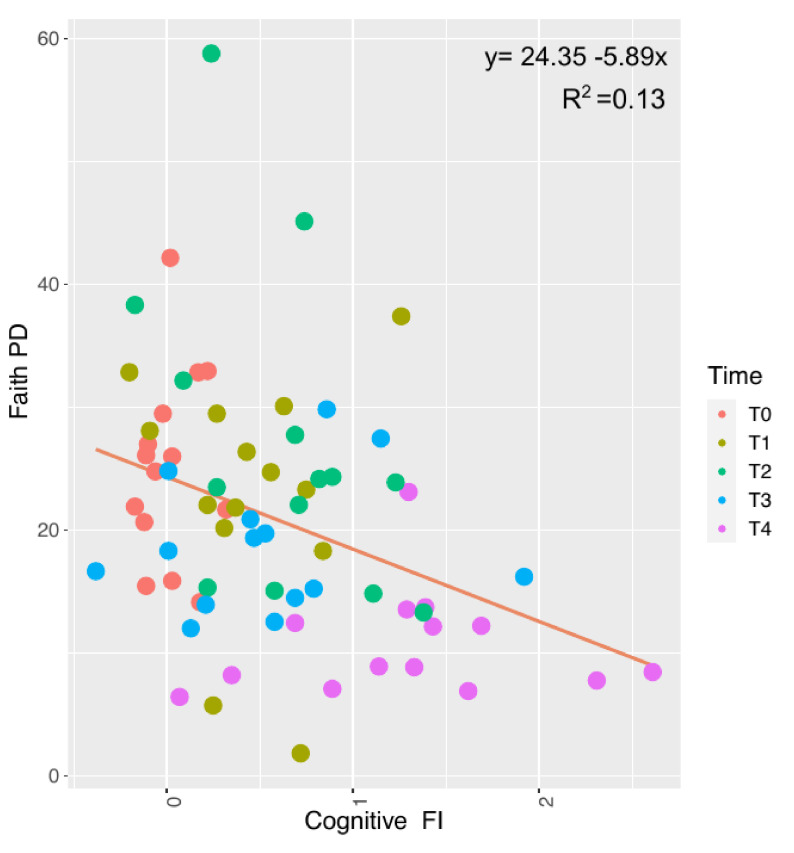
Figure plotting the correlation between Faith’s phylogenetic distance (PD) index and Cognitive FI. Reported is the linear regression equation used to fit experimental data. Colors in the graphs are reported according to the different experimental times as shown in the figure labels.

The next question to address was the possible identification of a frailty microbiome signature during aging. Therefore, we investigated the relationship between the Cognitive Frailty Index and the alpha diversity, evaluated by Shannon (see Supplementary Figure S4) and Faith’s PD indices (Figure 8). Faith’s PD index, but not Shannon index, showed a statistically significant relationship with the Cognitive FI in the performed linear models. In particular, in relation to an increase in the Cognitive FI value (coeff = 6.345, Figure 8, see Table S4 and Figure S4 in Supplementary Materials), a negative correlation in the estimated biodiversity occurred based on Faith’s PD index.

**Figure 9:**
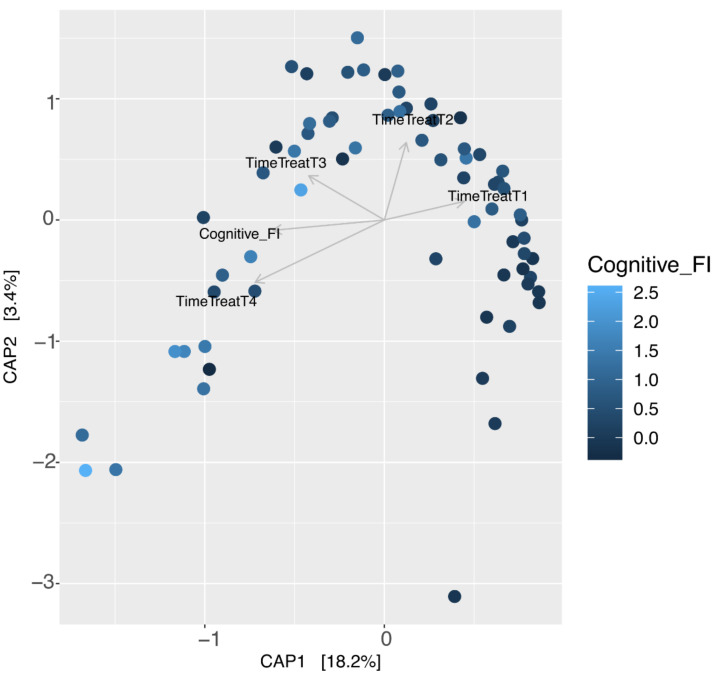
CAP analysis revealed that microbiomes varied by time, but with a slight effect on the Cognitive Frailty Index. CAP analysis was performed using the beta diversity based on Bray-Curtis distance metric constrained to time point and Cognitive FI. Each dot represents each sample’s coordinate on constrained PCoA1. Different levels of Cognitive FI are reported as a gradient scale (lower values in dark blue and higher values light blue).

This result was also supported by partial canonical analysis of principal coordinates (CAP analysis, Figure 9). The CAP scale revealed that an advanced state of senescence and a higher Cognitive FI value explained the largest proportion (18.2%, *p* < 0.001) of the variation in beta diversity (Figure 9).

However, the estimated LME models did not report a significant effect (*p* value > 0.05) of the Cognitive FI variation on the beta diversity of the microbiomes (see Table S5 in Supplementary Material), probably due to the low sample number.

**Figure 10:**
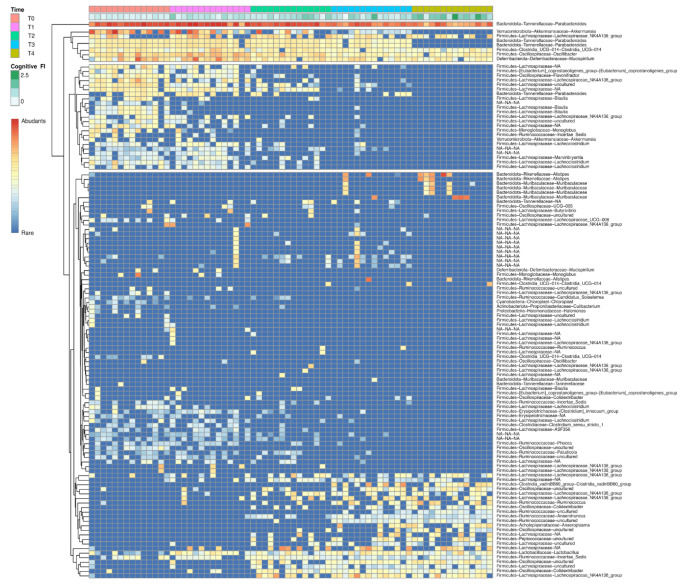
The heat map shows the distribution of the abundances of 218 ASVs whose variation was statistically significant in relation to the variation of the Cognitive FI. The analysis was performed using Deseq2 R package and the heat map was generated using phetamap R package. The variations in terms of abundance are indicated using the coloring scale in the legend. A green-white color scale is used to indicate the variation of Cognitive FI.

Furthermore, we applied the DESeq2 algorithm to test for differential abundance in bacterial groups that changed with respect to the Cognitive FI. We found 218 ASVs significantly linked to the variation in the Cognitive FI. Interestingly, the heat map returned different clusters (T0 and T1 versus T2, T3, and T4) in microbiome composition during the mice lifespan, confirming the beta diversity data (Figure 10).

These two identified clusters corresponding to microbiome composition in adulthood and senescence were specifically composed of some bacterial ASVs which were decreasing or that were increasing with physiological aging and frailty. In the discussion section, we address this point with specific comments about those ASVs specifically linked to the Cognitive FI change during the aging process.

## 4. Discussion

Declarative memory is one of the main features of the human personality, which is the capability to remember an object, a person, or a place previously encountered. The maintenance of the recognition memory capability of declarative memory has a great impact on the elderly social life. Animal and human studies have evidenced the so-called gut–brain axis, in which a crucial role is played by the gut microbiome through a dynamic, complex bidirectional relationship with the host, thus identifying the microbiota–gut–brain axis [15,42].

Elderly people display a different gut microbiome profile compared to healthy adults for multifactorial factors such as lifestyle, nutrition, lower motor capabilities, and reduced immune system functions [15]. Indeed, the gut microbiome is highly sensitive to environmental stimuli and its composition changes during the host lifespan. Animal models allow to minimize most of the differences in epigenetic factors such as, food, light, temperature, humidity, etc. A change in the gut microbiome composition during aging could be due both to a dysbiotic condition and/or to an adaptive mechanism trying to compensate for the dysbiosis. This longitudinal study aimed at investigating (i) the modifications in gut microbiome composition during aging; (ii) the relationship between gut microbiome composition and cognitive decline of recognition memory; and (iii) functional speculation about eubiotic and adaptive or dysbiotic and maladaptive changes during aging.

To achieve those goals, we monitored the gut microbiota composition and the Cognitive Frailty Index (FI) during aging [43]. Previously, we tuned an FI in mice for describing the aging decline of recognition memory by means of a battery of spontaneous behavioral tests that discriminate between the knowledge component and the recall component of the recognition memory [3,6].

The current longitudinal study demonstrated that the overall gut microbiome composition changed during the aging process. Nevertheless, as time went on, Cognitive FI increased, making it extremely difficult to isolate the effects of the two covariants (time and Cognitive FI) on overall gut microbiome composition (alpha and beta diversity levels). Despite this, we described a slight correlation between Cognitive FI and microbiomes (in terms of their taxonomic composition in the different mice examined), and this consideration was partly supported by the results of the CAP scale analysis. The not-significant effect of the LME model used for testing beta diversity in our experimental condition highlighted that it is not possible to use this variable as a predictor of total bacterial diversity. However, in a deeper taxonomic microbiome analysis, it was possible to identify some ASVs that specifically increased or decreased with Cognitive FI variation.

During aging, gut microbiome alpha diversity measures gave opposite results: the Shannon index increased, whereas Faith’s PD index decreased, in senescence. Shannon alpha diversity is sensitive to both the richness and the evenness (the total number of species and their relative abundance in the fecal sample). These data are in agreement with a recent paper [44], describing changes in human gut microbiome composition during aging also associated with healthy or unhealthy conditions.

Faith’s PD index represents the number of phylogenetic tree units and their distance within a sample. Therefore, our results suggest that during the aging process, we were witnessing an increase in the number of ASVs that were phylogenetically more similar in senescence compared to adulthood. We may speculate that, despite the high number of species in the aged mice, the similarity to the taxa level accounts for a lower flexibility from a functional point of view compared to young animals. Confirmation that aging significantly affected the overall microbiota composition also came from the inter-individual variation, estimated through the beta diversity, by a non-multidimensional scaling (NMDS) ordination approach. In fact, we identified five clusters, sequentially expressed and each one specific for each experimental time, thus creating a large gap between the adult and senescent mice microbiome.

The core phyla during aging were *Bacteoroidota* and *Firmicutes*: their relative abundance and ratio remained relatively stable throughout the lifespan. Regarding subcore phyla during aging, *Verrucomicrobia* disappeared between 20–21.5 months, matched by total disappearance of the *Akkermansiaceae* family and *Akkermansia* genus. A change in *Akkermansia*’s relative abundance was associated with metabolic diseases (i.e., diabetes, inflammatory bowel disease, and obesity) [45,46,47] but also with neurodegenerative diseases (i.e., Alzheimer’s and Parkinson’s diseases) [48,49,50]. At the gut level, *Akkermansia*, regulating the host immune response and reducing local inflammation, helps maintain the integrity of the gut barrier. Furthermore, *Akkermansia* influences the fat and sugar metabolism [47]. Recently, Ou et al. (2020) [51] and Higarza et al. (2021) [52] demonstrated that *Akkermansia* could improve cognitive performance in two different preclinical models. In particular, Ou et al. (2020) [51] found that the *Akkermansia* gavage, in addition to regulating the inflammation and sugar metabolism, significantly reduced the Aβ 40–42 levels in the cerebral cortex and improved the spatial and recognition memory of APP/PS1 mice, a model of Alzheimer’s disease (AD) [51]. Higarza et al. (2021) [52] demonstrated that *Akkermansia* gavage restored cognitive impairment related to nonalcoholic steatohepatitis in rats [52]. In parallel with the disappearance of *Akkermansiaciae*, we recorded the appearance of *Muribaculaceae* (previously known as S24-7). The *Muribaculaceae* family is one of the major utilizers of mucus-derived monosaccharides in the gut, contributing to SCFA production [53], in particular, propionate [54,55]. The SCFAs have a pivotal role in the host’s homeostasis and physiology, and SCFA concentrations are predictive of the host’s lifespan [54]. Indeed, the *Muribaculaceae* bacterial family was found as the dominant bacterial taxa in the gut of *Spalax leucodon*, an exceptional animal model of longevity [53]. Previous works have demonstrated a lower abundance of the *Muribaculaceae* family in aged mice compared to young animals, and the anti-aging intervention has been shown to partially reverse the gut microbiota composition of elderly mice, also increasing the relative abundance of the *Muribaculacea* [56,57]. We hypothesized that the increased *Muribaculaceae* relative abundance during physiological aging suggests a possible adaptive mechanism for the disappearance of the *Akkermansia,* trying to prevent a possible leaky gut. Next, we demonstrated a significant increase in the *Rikenellaceae* family during aging. Notably, in our samples, the *Alistipes* genus represented the entire *Rikenellaceae* family. Regarding this genus, conflicting data are reported [58], from a pathogenic role in cancer [59] to a preventative role in mental health, such as anxiety and depression [60].

Furthermore, we found a significant increase in *Clostridia vadinBB60* between adulthood and senescence. Recently, Juckel and colleagues (2021) [61], in patients with schizophrenia, suggested that an increase in the *Clostridia vadinBB60* group could be responsible for the neuroplasticity reduction in the central nervous system (CNS). Therefore, we supposed that the increase in *Clostridia vadinBB60* could exert a detrimental effect on cognitive performance during aging.

The relative abundance of some ASVs was significantly associated with Cognitive FI changes. In particular, ASVs belonging to *Parabacteroides, Clostridia UCG-014, Oscillibacter, Mucispirillum, Coprostanoligenes, Flavonifractor, Blautia, Monoglobus, Sedis, Lachnoclostridium, Oscillospiraceae_UCG_005, Marvinbryantia, Erysipelotrichaceae, Clostridium sensu stricto 1, Halomonas, Cutibacterium, Ruminococcaceae Candidatus Soleaferrea, Ruminococcaceae incertae sedis, Ruminococcaceae Paludicola, Ruminococcaceae Phocea*, and *Lachnospiraceae ASF356* significantly decreased in relation with the Cognitive FI increase. On the other hand, ASVs belonging to Ruminococcus, Colidextribacter, Anaerotruncus, Anaeroplasma, Peptococcaceae, Lactobacillus, and Ruminococcaceae incertae sedis increased in parallel to the Cognitive FI increment. We speculated that the increase in health-promoting or the decrease in pathobiont bacteria associated with the Cognitive FI increase could have an adaptive or compensatory role; on the contrary, the increase in unhealthy bacteria and a decrease in healthy bacteria with the FI increase could exert a dysbiotic role (Table 3).

Furthermore, about the other ASVs which decreased or increased, not yet presently described in the present study, any previous literature data are available, to the best of our knowledge, regarding their relationship with both aging and cognitive frailty.

In summary, our data demonstrated that aging significantly affected the overall gut microbiome composition in a complex way, increasing the number of ASVs and decreasing the phylogenetic distance among them. Beta diversity confirmed the presence of different clusters between adult and senescent mice. Cognitive frailty was associated with some specific changes in ASVs. We proposed that some of those ASV modifications could be a challenge in the eubiotic and adaptive direction and the opposite in the dysbiotic and maladaptive direction. The inverse correlation between Faith’s PD index and cognitive frailty suggests that cognitive decline is accompanied by a shrinkage of the gut microbiome functionality. Furthermore, this potential plasticity in the gut microbiome composition paves the way to the use of psychobiotics for prevention of cognitive frailty during aging [131].

## Figures and Tables

**Table 1:** Statistical analysis of the effect of aging on alpha diversity based on Shannon diversity index (on the top) and Faith’s phylogenetic distance (on the bottom).

	Shannon Index	
Combination	WSRT	FDR *p*-Value
T0–T1	17	0.092
T1–T2	6	0.007
T2–T3	13	0.011
T3–T4	41	0.787
**Faith’s PD Index**		
**Combination**	**WSRT**	**FDR *p*-Value**
T0–T1	36	0.850
T1–T2	31	0.569
T2–T3	24	0.0785
T3–T4	0	0.0002

**Table 2:** Statistical analysis of the effect of aging on beta diversity based on Bray-Curtis distance matrix.

**Combination**	**SumsOfSqs**	**MeanSqs**	**F.Model**	**R^2^**	***p*** **-Value. Corrected**
T0–T1	0.191	0.191	2.200	0.084	0.029
T1–T2	0.205	0.205	2.317	0.088	0.004
T1–T3	0.746	0.746	6.188	0.205	0.001
T2–T3	0.351	0.351	2.516	0.088	0.001
T3–T4	0.301	0.301	1.555	0.059	0.109

**Table 3:** Proposed dysbiotic or adaptive roles of gut bacteria that significantly changed with Cognitive Frailty Index. We searched in the literature the involvement of selected bacteria on host health, and in particular, on CNS.

Genera	Published Effects on Host Health	Changes with Cognitive Frailty Increase	Possible Role
*Parabacteroides*	Dichotomous role demonstrated also in CNS [62,63,64].	Decrease	Dysbiotic/Adaptive
*Clostridia UCG-014*	Pro-inflammatory [65,66,67].	Decrease	Adaptive
*Oscillibacter*	Negatively related to cognitive performance [68]; related to depression [60,69], metabolic disease [70], and inflammation [71].	Decrease	Adaptive
*Mucispirillum*	Opportunistic pathogens [72]; positively related to inflammation, LPS [72,73,74], and changes in different behavioral domains [75].	Decrease	Dysbiotic
*Coprostanoligenes*	Negatively related to inflammation [76,77,78].	Decrease	Dysbiotic
*Flavonifractor*	Dichotomous role, also demonstrated in CNS [79,80,81,82].	Decrease	Dysbiotic/Adaptive
*Blautia*	Butyrate producer with probiotic potential [83].	Decrease	Dysbiotic
*Monoglobus*	Negatively related to amyloid presence in the brain [84].	Decrease	Dysbiotic
*Lachnoclostridium*	Butyrate producer [85,86].	Decrease	Dysbiotic
*Oscillospiraceae UCG_005*	Negatively related to inflammation [81].	Decrease	Dysbiotic
*Marvinbryantia*	Negatively related to amyloid presence in the brain [84].	Decrease	Dysbiotic
*Erysipelotrichaceae*	Pro-inflammatory [87,88,89]; enriched in mouse AD model and senescence-accelerated mice model [87,89].	Decrease	Adaptive
*Clostridium sensu stricto 1*, known also as *C. butyricum* [90]	Dichotomous role [91,92,93,94,95].	Decrease	Dysbiotic/Adaptive
*Halomonas*	Dichotomous role [96,97].	Decrease	Dysbiotic/Adaptive
*Ruminococcus*	Pro-inflammatory [98,99,100]; dysregulated in patients with depression, physical frailty and sarcopenia, AD, and PD and MCI [80,101,102,103,104]; risk indicator of MCI [105]; negatively related to cognitive performance [103,104,105] and to marker of neuronal health [106].	Increase	Dysbiotic
*Colidextribacter*	Positively related to inflammation [65,107]; decreased in PD patients [108].	Increase	Dysbiotic
*Anaerotruncus*	Dichotomous role [109,110,111,112], also in CNS (AD, PD, and cognitive impairment) [109,113,114].	Increase	Dysbiotic/Adaptive
*Anaeroplasma*	Healthy bacterium [115] decreased in AD mice model [116] and in obese rats [117]; negatively related to inflammation [118].	Increase	Adaptive
*Peptococcaceae*	Pro-inflammatory [118,119].	Increase	Dysbiotic
*Lactobacillus*	Anti-inflammatory, butyrate producer with probiotic effect [120,121,122], its beneficial effects are demonstrated also in CNS (altered behaviors, depression, anxiety, stress, cognitive impairment, andAD models) [123,124,125,126,127,128]; reduced sarcopenic process and physical frailty in mice [129].	Increase	Adaptive
*Ruminococcaceae incertae sedis*	Anti-inflammatory, butyrate producer [130].	Increase	Adaptive

## Data Availability

The sequences that were generated in this study have been deposited into the EMBL-EBI database in PRJEB54046 project.

## Supplementary Materials

The following supporting information can be downloaded at: https://www.mdpi.com/article/10.3390/nu14142937/s1, Figure S1: Rarefaction curve; Figure S2: Number of reads; Figure S3: Simpson index; Figure S4: correlation between Shannon index and Cognitive FI. Table S1: Statistical analysis of the effect of aging on alpha diversity based on Shannon diversity index (on the top) and Faith’s phylogenetic distance (on the bottom), including the comparison among all time points; Table S2: Statistical analysis of the effect of aging on beta diversity based on Bray-Curtis distance matrix, including the comparison among all time points; Table S3: LME used to estimate the effect of time on Cognitive FI; Table S4: LME used to estimate the effect of time and Cognitive FI on Faith’s PD index; Table S5: LME used to estimate the effect of time and Cognitive FI on the beta diversity, based on Bray-Curtis index.

## References

[1] López-Otín C., Blasco M.A., Partridge L., Serrano M., Kroemer G. (2013). The Hallmarks of Aging. Cell.

[2] Bana B., Cabreiro F. (2019). The Microbiome and Aging. Annu. Rev. Genet..

[3] Ratto D., Corana F., Mannucci B., Priori E.C., Cobelli F., Roda E., Ferrari B., Occhinegro A., Di Iorio C., De Luca F. (2019). Hericium Erinaceus Improves Recognition Memory and Induces Hippocampal and Cerebellar Neurogenesis in Frail Mice during Aging. Nutrients.

[4] Burke S.N., Ryan L., Barnes C.A. (2012). Characterizing Cognitive Aging of Recognition Memory and Related Processes in Animal Models and in Humans. Front. Aging Neurosci..

[5] Turriziani P., Serra L., Fadda L., Caltagirone C., Carlesimo G.A. (2008). Recollection and Familiarity in Hippocampal Amnesia. Hippocampus.

[6] Rossi P., Cesaroni V., Brandalise F., Occhinegro A., Ratto D., Perrucci F., Lanaia V., Girometta C., Orrù G., Savino E. (2018). Dietary Supplementation of Lion’s Mane Medicinal Mushroom, *Hericium erinaceus* (Agaricomycetes), and Spatial Memory in Wild-Type Mice. Int. J. Med. Mushrooms.

[7] Cesari M., Calvani R., Marzetti E. (2017). Frailty in Older Persons. Clin. Geriatr. Med..

[8] Fried L.P., Tangen C.M., Walston J., Newman A.B., Hirsch C., Gottdiener J., Seeman T., Tracy R., Kop W.J., Burke G. (2001). Frailty in Older Adults: Evidence for a Phenotype. J. Gerontol. A Biol. Sci. Med. Sci..

[9] Bandeen-Roche K., Xue Q.-L., Ferrucci L., Walston J., Guralnik J.M., Chaves P., Zeger S.L., Fried L.P. (2006). Phenotype of Frailty: Characterization in the Women’s Health and Aging Studies. J. Gerontol. A Biol. Sci. Med. Sci..

[10] Piggott D.A., Tuddenham S. (2020). The Gut Microbiome and Frailty. Transl. Res..

[11] Gomez-Cabrera M.C., Garcia-Valles R., Rodriguez-Mañas L., Garcia-Garcia F.J., Olaso-Gonzalez G., Salvador-Pascual A., Tarazona-Santabalbina F.J., Viña J. (2017). A New Frailty Score for Experimental Animals Based on the Clinical Phenotype: Inactivity as a Model of Frailty. J. Gerontol. A Biol. Sci. Med. Sci..

[12] Arai H., Satake S., Kozaki K. (2018). Cognitive Frailty in Geriatrics. Clin. Geriatr. Med..

[13] Hernandez-Segura A., Nehme J., Demaria M. (2018). Hallmarks of Cellular Senescence. Trends Cell. Biol..

[14] Wen J., Wang Y., Yuan M., Huang Z., Zou Q., Pu Y., Zhao B., Cai Z. (2021). Role of Mismatch Repair in Aging. Int. J. Biol. Sci..

[15] Nagpal R., Mainali R., Ahmadi S., Wang S., Singh R., Kavanagh K., Kitzman D.W., Kushugulova A., Marotta F., Yadav H. (2018). Gut Microbiome and Aging: Physiological and Mechanistic Insights. Nutr. Healthy Aging.

[16] DeJong E.N., Surette M.G., Bowdish D.M.E. (2020). The Gut Microbiota and Unhealthy Aging: Disentangling Cause from Consequence. Cell Host Microbe.

[17] Guarner F., Malagelada J.-R. (2003). Gut Flora in Health and Disease. Lancet.

[18] Jandhyala S.M., Talukdar R., Subramanyam C., Vuyyuru H., Sasikala M., Nageshwar Reddy D. (2015). Role of the Normal Gut Microbiota. World J. Gastroenterol..

[19] Shortt C., Hasselwander O., Meynier A., Nauta A., Fernández E.N., Putz P., Rowland I., Swann J., Türk J., Vermeiren J. (2018). Systematic Review of the Effects of the Intestinal Microbiota on Selected Nutrients and Non-Nutrients. Eur. J. Nutr..

[20] Bäckhed F., Ley R.E., Sonnenburg J.L., Peterson D.A., Gordon J.I. (2005). Host-Bacterial Mutualism in the Human Intestine. Science.

[21] Turnbaugh P.J., Ley R.E., Hamady M., Fraser-Liggett C.M., Knight R., Gordon J.I. (2007). The Human Microbiome Project. Nature.

[22] Quigley E.M.M. (2013). Gut Bacteria in Health and Disease. Gastroenterol. Hepatol..

[23] Clemente J.C., Ursell L.K., Parfrey L.W., Knight R. (2012). The Impact of the Gut Microbiota on Human Health: An Integrative View. Cell.

[24] La Rosa F., Clerici M., Ratto D., Occhinegro A., Licito A., Romeo M., Iorio C.D., Rossi P. (2018). The Gut-Brain Axis in Alzheimer’s Disease and Omega-3. A Critical Overview of Clinical Trials. Nutrients.

[25] Marizzoni M., Provasi S., Cattaneo A., Frisoni G.B. (2017). Microbiota and Neurodegenerative Diseases. Curr. Opin. Neurol..

[26] Ticinesi A., Tana C., Nouvenne A., Prati B., Lauretani F., Meschi T. (2018). Gut Microbiota, Cognitive Frailty and Dementia in Older Individuals: A Systematic Review. Clin. Interv. Aging.

[27] Brandalise F., Cesaroni V., Gregori A., Repetti M., Romano C., Orrù G., Botta L., Girometta C., Guglielminetti M.L., Savino E. (2017). Dietary Supplementation of Hericium Erinaceus Increases Mossy Fiber-CA3 Hippocampal Neurotransmission and Recognition Memory in Wild-Type Mice. Evid. Based Complement. Alternat. Med..

[28] Bolyen E., Rideout J.R., Dillon M.R., Bokulich N.A., Abnet C.C., Al-Ghalith G.A., Alexander H., Alm E.J., Arumugam M., Asnicar F. (2019). Reproducible, Interactive, Scalable and Extensible Microbiome Data Science Using QIIME 2. Nat. Biotechnol..

[29] Callahan B.J., McMurdie P.J., Rosen M.J., Han A.W., Johnson A.J.A., Holmes S.P. (2016). DADA2: High-Resolution Sample Inference from Illumina Amplicon Data. Nat. Methods.

[30] Bokulich N.A., Kaehler B.D., Rideout J.R., Dillon M., Bolyen E., Knight R., Huttley G.A., Gregory Caporaso J. (2018). Optimizing Taxonomic Classification of Marker-Gene Amplicon Sequences with QIIME 2’s Q2-Feature-Classifier Plugin. Microbiome.

[31] Quast C., Pruesse E., Yilmaz P., Gerken J., Schweer T., Yarza P., Peplies J., Glöckner F.O. (2013). The SILVA Ribosomal RNA Gene Database Project: Improved Data Processing and Web-Based Tools. Nucleic Acids Res..

[32] McMurdie P.J., Holmes S. (2013). Phyloseq: An R Package for Reproducible Interactive Analysis and Graphics of Microbiome Census Data. PLoS ONE.

[33] Faith D.P. (2006). Science and Philosophy for Molecular Systematics: Which Is the Cart and Which Is the Horse?. Mol. Phylogenet. Evol..

[34] Cuzick J. (1985). A Wilcoxon-Type Test for Trend. Stat. Med..

[35] Vegan: Community Ecology Package. R Package Vegan, Vers. 2.2-1. https://www.worldagroforestry.org/publication/vegan-community-ecology-package-r-package-vegan-vers-22-1.

[36] Anderson M.J., Willis T.J. (2003). Canonical Analysis of Principal Coordinates: A Useful Method of Constrained Ordination for Ecology. Ecology.

[37] Kruskal J.B. (1964). Multidimensional Scaling by Optimizing Goodness of Fit to a Nonmetric Hypothesis. Psychometrika.

[38] Love M.I., Huber W., Anders S. (2014). Moderated Estimation of Fold Change and Dispersion for RNA-Seq Data with DESeq2. Genome Biol..

[39] Foster Z.S.L., Sharpton T.J., Grünwald N.J. (2017). Metacoder: An R Package for Visualization and Manipulation of Community Taxonomic Diversity Data. PLoS Comput. Biol..

[40] Ggplot2: Create Elegant Data Visualisations Using the Grammar of Graphics—Ggplot2-Package. https://ggplot2.tidyverse.org/reference/ggplot2-package.html.

[41] Fox J., Weisberg S. (2011). An R Companion to Applied Regression.

[42] O’Toole P.W., Jeffery I.B. (2015). Gut Microbiota and Aging. Science.

[43] Santoro A., Ostan R., Candela M., Biagi E., Brigidi P., Capri M., Franceschi C. (2018). Gut Microbiota Changes in the Extreme Decades of Human Life: A Focus on Centenarians. Cell Mol. Life Sci..

[44] Wilmanski T., Diener C., Rappaport N., Patwardhan S., Wiedrick J., Lapidus J., Earls J.C., Zimmer A., Glusman G., Robinson M. (2021). Gut Microbiome Pattern Reflects Healthy Ageing and Predicts Survival in Humans. Nat. Metab..

[45] Derrien M., Belzer C., de Vos W.M. (2017). *Akkermansia muciniphila* and Its Role in Regulating Host Functions. Microb. Pathog..

[46] Naito Y., Uchiyama K., Takagi T. (2018). A Next-Generation Beneficial Microbe: *Akkermansia muciniphila*. J. Clin. Biochem. Nutr..

[47] Macchione I.G., Lopetuso L.R., Ianiro G., Napoli M., Gibiino G., Rizzatti G., Petito V., Gasbarrini A., Scaldaferri F. (2019). *Akkermansia muciniphila*: Key Player in Metabolic and Gastrointestinal Disorders. Eur. Rev. Med. Pharmacol. Sci..

[48] Gerhardt S., Mohajeri M.H. (2018). Changes of Colonic Bacterial Composition in Parkinson’s Disease and Other Neurodegenerative Diseases. Nutrients.

[49] Heintz-Buschart A., Pandey U., Wicke T., Sixel-Döring F., Janzen A., Sittig-Wiegand E., Trenkwalder C., Oertel W.H., Mollenhauer B., Wilmes P. (2018). The Nasal and Gut Microbiome in Parkinson’s Disease and Idiopathic Rapid Eye Movement Sleep Behavior Disorder. Mov. Disord..

[50] Nishiwaki H., Ito M., Ishida T., Hamaguchi T., Maeda T., Kashihara K., Tsuboi Y., Ueyama J., Shimamura T., Mori H. (2020). Meta-Analysis of Gut Dysbiosis in Parkinson’s Disease. Mov. Disord..

[51] Ou Z., Deng L., Lu Z., Wu F., Liu W., Huang D., Peng Y. (2020). Protective Effects of *Akkermansia muciniphila* on Cognitive Deficits and Amyloid Pathology in a Mouse Model of Alzheimer’s Disease. Nutr. Diabetes.

[52] Higarza S.G., Arboleya S., Arias J.L., Gueimonde M., Arias N. (2021). *Akkermansia muciniphila* and Environmental Enrichment Reverse Cognitive Impairment Associated with High-Fat High-Cholesterol Consumption in Rats. Gut Microbes.

[53] Sibai M., Altuntaş E., Yıldırım B., Öztürk G., Yıldırım S., Demircan T. (2020). Microbiome and Longevity: High Abundance of Longevity-Linked Muribaculaceae in the Gut of the Long-Living Rodent Spalax Leucodon. OMICS.

[54] Smith B.J., Miller R.A., Ericsson A.C., Harrison D.C., Strong R., Schmidt T.M. (2019). Changes in the Gut Microbiome and Fermentation Products Concurrent with Enhanced Longevity in Acarbose-Treated Mice. BMC Microbiol..

[55] Pereira F.C., Wasmund K., Cobankovic I., Jehmlich N., Herbold C.W., Lee K.S., Sziranyi B., Vesely C., Decker T., Stocker R. (2020). Rational Design of a Microbial Consortium of Mucosal Sugar Utilizers Reduces Clostridiodes Difficile Colonization. Nat. Commun..

[56] Han D., Li Z., Liu T., Yang N., Li Y., He J., Qian M., Kuang Z., Zhang W., Ni C. (2020). Prebiotics Regulation of Intestinal Microbiota Attenuates Cognitive Dysfunction Induced by Surgery Stimulation in APP/PS1 Mice. Aging Dis..

[57] Shenghua P., Ziqin Z., Shuyu T., Huixia Z., Xianglu R., Jiao G. (2020). An Integrated Fecal Microbiome and Metabolome in the Aged Mice Reveal Anti-Aging Effects from the Intestines and Biochemical Mechanism of FuFang Zhenshu TiaoZhi(FTZ). Biomed. Pharmacother..

[58] Parker B.J., Wearsch P.A., Veloo A.C.M., Rodriguez-Palacios A. (2020). The Genus Alistipes: Gut Bacteria With Emerging Implications to Inflammation, Cancer, and Mental Health. Front. Immunol..

[59] Yang Y., Jobin C. (2017). Novel Insights into Microbiome in Colitis and Colorectal Cancer. Curr. Opin. Gastroenterol..

[60] Naseribafrouei A., Hestad K., Avershina E., Sekelja M., Linløkken A., Wilson R., Rudi K. (2014). Correlation between the Human Fecal Microbiota and Depression. Neurogastroenterol. Motil..

[61] Juckel G., Manitz M.-P., Freund N., Gatermann S. (2021). Impact of Poly I:C Induced Maternal Immune Activation on Offspring’s Gut Microbiome Diversity—Implications for Schizophrenia. Prog. Neuropsychopharmacol. Biol. Psychiatry.

[62] Ezeji J.C., Sarikonda D.K., Hopperton A., Erkkila H.L., Cohen D.E., Martinez S.P., Cominelli F., Kuwahara T., Dichosa A.E.K., Good C.E. (2021). Parabacteroides Distasonis: Intriguing Aerotolerant Gut Anaerobe with Emerging Antimicrobial Resistance and Pathogenic and Probiotic Roles in Human Health. Gut Microbes.

[63] Wang K., Liao M., Zhou N., Bao L., Ma K., Zheng Z., Wang Y., Liu C., Wang W., Wang J. (2019). Parabacteroides Distasonis Alleviates Obesity and Metabolic Dysfunctions via Production of Succinate and Secondary Bile Acids. Cell Rep..

[64] Chang C.-J., Lin T.-L., Tsai Y.-L., Wu T.-R., Lai W.-F., Lu C.-C., Lai H.-C. (2019). Next Generation Probiotics in Disease Amelioration. J. Food Drug Anal..

[65] Wang Y., Nan X., Zhao Y., Jiang L., Wang H., Zhang F., Hua D., Liu J., Yao J., Yang L. (2021). Dietary Supplementation of Inulin Ameliorates Subclinical Mastitis via Regulation of Rumen Microbial Community and Metabolites in Dairy Cows. Microbiol. Spectr..

[66] Liu Y., Zhou M., Yang M., Jin C., Song Y., Chen J., Gao M., Ai Z., Su D. (2021). Pulsatilla Chinensis Saponins Ameliorate Inflammation and DSS-Induced Ulcerative Colitis in Rats by Regulating the Composition and Diversity of Intestinal Flora. Front. Cell Infect. Microbiol..

[67] Zhao J.-D., Li Y., Sun M., Yu C.-J., Li J.-Y., Wang S.-H., Yang D., Guo C.-L., Du X., Zhang W.-J. (2021). Effect of Berberine on Hyperglycaemia and Gut Microbiota Composition in Type 2 Diabetic Goto-Kakizaki Rats. World J. Gastroenterol..

[68] Lee D.-Y., Shin Y.-J., Kim J.-K., Jang H.-M., Joo M.-K., Kim D.-H. (2021). Alleviation of Cognitive Impairment by Gut Microbiota Lipopolysaccharide Production-Suppressing *Lactobacillus plantarum* and *Bifidobacterium longum* in Mice. Food Funct..

[69] Barandouzi Z.A., Starkweather A.R., Henderson W.A., Gyamfi A., Cong X.S. (2020). Altered Composition of Gut Microbiota in Depression: A Systematic Review. Front. Psychiatry.

[70] Thingholm L.B., Rühlemann M.C., Koch M., Fuqua B., Laucke G., Boehm R., Bang C., Franzosa E.A., Hübenthal M., Rahnavard A. (2019). Obese Individuals with and without Type 2 Diabetes Show Different Gut Microbial Functional Capacity and Composition. Cell Host Microbe.

[71] Tran T.T.T., Cousin F.J., Lynch D.B., Menon R., Brulc J., Brown J.R.-M., O’Herlihy E., Butto L.F., Power K., Jeffery I.B. (2019). Prebiotic Supplementation in Frail Older People Affects Specific Gut Microbiota Taxa but Not Global Diversity. Microbiome.

[72] Wei J., Zhao Y., Zhou C., Zhao Q., Zhong H., Zhu X., Fu T., Pan L., Shang Q., Yu G. (2021). Dietary Polysaccharide from Enteromorpha Clathrata Attenuates Obesity and Increases the Intestinal Abundance of Butyrate-Producing Bacterium, *Eubacterium xylanophilum*, in Mice Fed a High-Fat Diet. Polymers.

[73] Li K., Zhang L., Xue J., Yang X., Dong X., Sha L., Lei H., Zhang X., Zhu L., Wang Z. (2019). Dietary Inulin Alleviates Diverse Stages of Type 2 Diabetes Mellitus via Anti-Inflammation and Modulating Gut Microbiota in Db/Db Mice. Food Funct..

[74] Shintouo C.M., Mets T., Beckwee D., Bautmans I., Ghogomu S.M., Souopgui J., Leemans L., Meriki H.D., Njemini R. (2020). Is Inflammageing Influenced by the Microbiota in the Aged Gut? A Systematic Review. Exp. Gerontol..

[75] Lyu Z., Ghoshdastidar S., Rekha K.R., Suresh D., Mao J., Bivens N., Kannan R., Joshi T., Rosenfeld C.S., Upendran A. (2021). Developmental Exposure to Silver Nanoparticles Leads to Long Term Gut Dysbiosis and Neurobehavioral Alterations. Sci. Rep..

[76] Ren D., Li L., Schwabacher A.W., Young J.W., Beitz D.C. (1996). Mechanism of Cholesterol Reduction to Coprostanol by Eubacterium Coprostanoligenes ATCC 51222. Steroids.

[77] Liu R.T., Rowan-Nash A.D., Sheehan A.E., Walsh R.F.L., Sanzari C.M., Korry B.J., Belenky P. (2020). Reductions in Anti-Inflammatory Gut Bacteria Are Associated with Depression in a Sample of Young Adults. Brain Behav. Immun..

[78] Bai D., Sun T., Zhao J., Du J., Bu X., Cao W., Zhao Y., Lu N. (2021). Oroxylin A Maintains the Colonic Mucus Barrier to Reduce Disease Susceptibility by Reconstituting a Dietary Fiber-Deprived Gut Microbiota. Cancer Lett..

[79] Arias-Jayo N., Abecia L., Alonso-Sáez L., Ramirez-Garcia A., Rodriguez A., Pardo M.A. (2018). High-Fat Diet Consumption Induces Microbiota Dysbiosis and Intestinal Inflammation in Zebrafish. Microb. Ecol..

[80] Jiang H., Ling Z., Zhang Y., Mao H., Ma Z., Yin Y., Wang W., Tang W., Tan Z., Shi J. (2015). Altered Fecal Microbiota Composition in Patients with Major Depressive Disorder. Brain Behav. Immun..

[81] Zhang Q., Yun Y., An H., Zhao W., Ma T., Wang Z., Yang F. (2022). Gut Microbiome and Daytime Function in Chinese Patients with Major Depressive Disorder. J. Psychosom. Res..

[82] Mikami A., Ogita T., Namai F., Shigemori S., Sato T., Shimosato T. (2020). Oral Administration of Flavonifractor Plautii, a Bacteria Increased With Green Tea Consumption, Promotes Recovery From Acute Colitis in Mice via Suppression of IL-17. Front. Nutr..

[83] Liu X., Mao B., Gu J., Wu J., Cui S., Wang G., Zhao J., Zhang H., Chen W. (2021). Blautia-a New Functional Genus with Potential Probiotic Properties?. Gut Microbes.

[84] Verhaar B.J.H., Hendriksen H.M.A., de Leeuw F.A., Doorduijn A.S., van Leeuwenstijn M., Teunissen C.E., Barkhof F., Scheltens P., Kraaij R., van Duijn C.M. (2021). Gut Microbiota Composition Is Related to AD Pathology. Front. Immunol..

[85] Yutin N., Galperin M.Y. (2013). A Genomic Update on Clostridial Phylogeny: Gram-Negative Spore Formers and Other Misplaced Clostridia. Environ. Microbiol..

[86] Dandachi I., Anani H., Hadjadj L., Brahimi S., Lagier J.-C., Daoud Z., Rolain J.-M. (2021). Genome Analysis of Lachnoclostridium Phocaeense Isolated from a Patient after Kidney Transplantation in Marseille. New Microbes New Infect..

[87] Chen L.-H., Wang M.-F., Chang C.-C., Huang S.-Y., Pan C.-H., Yeh Y.-T., Huang C.-H., Chan C.-H., Huang H.-Y. (2021). Lacticaseibacillus Paracasei PS23 Effectively Modulates Gut Microbiota Composition and Improves Gastrointestinal Function in Aged SAMP8 Mice. Nutrients.

[88] Jena P.K., Sheng L., Nguyen M., Di Lucente J., Hu Y., Li Y., Maezawa I., Jin L.-W., Wan Y.-J.Y. (2020). Dysregulated Bile Acid Receptor-Mediated Signaling and IL-17A Induction Are Implicated in Diet-Associated Hepatic Health and Cognitive Function. Biomark Res..

[89] Bäuerl C., Collado M.C., Diaz Cuevas A., Viña J., Pérez Martínez G. (2018). Shifts in Gut Microbiota Composition in an APP/PSS1 Transgenic Mouse Model of Alzheimer’s Disease during Lifespan. Lett. Appl. Microbiol..

[90] Lawson P.A., Citron D.M., Tyrrell K.L., Finegold S.M. (2016). Reclassification of Clostridium Difficile as Clostridioides Difficile (Hall and O’Toole 1935) Prévot 1938. Anaerobe.

[91] Dürre P. (2014). Physiology and Sporulation in Clostridium. Microbiol. Spectr..

[92] Zhao Q., Yang W.-R., Wang X.-H., Li G.-Q., Xu L.-Q., Cui X., Liu Y., Zuo X.-L. (2019). Clostridium Butyricum Alleviates Intestinal Low-Grade Inflammation in TNBS-Induced Irritable Bowel Syndrome in Mice by Regulating Functional Status of Lamina Propria Dendritic Cells. World J. Gastroenterol..

[93] Kashiwagi I., Morita R., Schichita T., Komai K., Saeki K., Matsumoto M., Takeda K., Nomura M., Hayashi A., Kanai T. (2015). Smad2 and Smad3 Inversely Regulate TGF-β Autoinduction in Clostridium Butyricum-Activated Dendritic Cells. Immunity.

[94] Rom O., Liu Y., Liu Z., Zhao Y., Wu J., Ghrayeb A., Villacorta L., Fan Y., Chang L., Wang L. (2020). Glycine-Based Treatment Ameliorates NAFLD by Modulating Fatty Acid Oxidation, Glutathione Synthesis, and the Gut Microbiome. Sci. Transl. Med..

[95] Zeng Q., Li D., He Y., Li Y., Yang Z., Zhao X., Liu Y., Wang Y., Sun J., Feng X. (2019). Discrepant Gut Microbiota Markers for the Classification of Obesity-Related Metabolic Abnormalities. Sci. Rep..

[96] Franco-de-Moraes A.C., de Almeida-Pititto B., da Rocha Fernandes G., Gomes E.P., da Costa Pereira A., Ferreira S.R.G. (2017). Worse Inflammatory Profile in Omnivores than in Vegetarians Associates with the Gut Microbiota Composition. Diabetol. Metab. Syndr..

[97] Ialenti A., Di Meglio P., Grassia G., Maffia P., Di Rosa M., Lanzetta R., Molinaro A., Silipo A., Grant W., Ianaro A. (2006). A Novel Lipid A from Halomonas Magadiensis Inhibits Enteric LPS-Induced Human Monocyte Activation. Eur. J. Immunol..

[98] Van Soest A.P.M., Hermes G.D.A., Berendsen A.A.M., van de Rest O., Zoetendal E.G., Fuentes S., Santoro A., Franceschi C., de Groot L.C.P.G.M., de Vos W.M. (2020). Associations between Pro- and Anti-Inflammatory Gastro-Intestinal Microbiota, Diet, and Cognitive Functioning in Dutch Healthy Older Adults: The NU-AGE Study. Nutrients.

[99] Henke M.T., Kenny D.J., Cassilly C.D., Vlamakis H., Xavier R.J., Clardy J. (2019). Ruminococcus Gnavus, a Member of the Human Gut Microbiome Associated with Crohn’s Disease, Produces an Inflammatory Polysaccharide. Proc. Natl. Acad. Sci. USA.

[100] Beaud D., Tailliez P., Anba-Mondoloni J. (2005). Genetic Characterization of the Beta-Glucuronidase Enzyme from a Human Intestinal Bacterium, Ruminococcus Gnavus. Microbiology.

[101] Chahwan B., Kwan S., Isik A., van Hemert S., Burke C., Roberts L. (2019). Gut Feelings: A Randomised, Triple-Blind, Placebo-Controlled Trial of Probiotics for Depressive Symptoms. J. Affect. Disord..

[102] Picca A., Ponziani F.R., Calvani R., Marini F., Biancolillo A., Coelho-Junior H.J., Gervasoni J., Primiano A., Putignani L., Del Chierico F. (2019). Gut Microbial, Inflammatory and Metabolic Signatures in Older People with Physical Frailty and Sarcopenia: Results from the BIOSPHERE Study. Nutrients.

[103] Zhuang Z.-Q., Shen L.-L., Li W.-W., Fu X., Zeng F., Gui L., Lü Y., Cai M., Zhu C., Tan Y.-L. (2018). Gut Microbiota Is Altered in Patients with Alzheimer’s Disease. J. Alzheimers Dis..

[104] Ren T., Gao Y., Qiu Y., Jiang S., Zhang Q., Zhang J., Wang L., Zhang Y., Wang L., Nie K. (2020). Gut Microbiota Altered in Mild Cognitive Impairment Compared With Normal Cognition in Sporadic Parkinson’s Disease. Front. Neurol..

[105] Khine W.W.T., Voong M.L., Ng T.K.S., Feng L., Rane G.A., Kumar A.P., Kua E.H., Mahendran R., Mahendran R., Lee Y.-K. (2020). Mental Awareness Improved Mild Cognitive Impairment and Modulated Gut Microbiome. Aging.

[106] Mudd A.T., Berding K., Wang M., Donovan S.M., Dilger R.N. (2017). Serum Cortisol Mediates the Relationship between Fecal Ruminococcus and Brain N-Acetylaspartate in the Young Pig. Gut Microbes.

[107] Duan R., Guan X., Huang K., Zhang Y., Li S., Xia J., Shen M. (2021). Flavonoids from Whole-Grain Oat Alleviated High-Fat Diet-Induced Hyperlipidemia via Regulating Bile Acid Metabolism and Gut Microbiota in Mice. J. Agric. Food Chem..

[108] Kenna J.E., Chua E.G., Bakeberg M., Tay A., McGregor S., Gorecki A., Horne M., Marshall B., Mastaglia F.L., Anderton R.S. (2021). Changes in the Gut Microbiome and Predicted Functional Metabolic Effects in an Australian Parkinson’s Disease Cohort. Front. Neurosci..

[109] Xu M., Mo X., Huang H., Chen X., Liu H., Peng Z., Chen L., Rong S., Yang W., Xu S. (2020). Yeast β-Glucan Alleviates Cognitive Deficit by Regulating Gut Microbiota and Metabolites in Aβ1-42-Induced AD-like Mice. Int. J. Biol. Macromol..

[110] Kong C., Gao R., Yan X., Huang L., Qin H. (2019). Probiotics Improve Gut Microbiota Dysbiosis in Obese Mice Fed a High-Fat or High-Sucrose Diet. Nutrition.

[111] El Aidy S., Van den Abbeele P., Van de Wiele T., Louis P., Kleerebezem M. (2013). Intestinal Colonization: How Key Microbial Players Become Established in This Dynamic Process: Microbial Metabolic Activities and the Interplay between the Host and Microbes. Bioessays.

[112] Wang J., Ji H., Wang S., Liu H., Zhang W., Zhang D., Wang Y. (2018). Probiotic *Lactobacillus plantarum* Promotes Intestinal Barrier Function by Strengthening the Epithelium and Modulating Gut Microbiota. Front. Microbiol..

[113] Jeong S., Huang L.-K., Tsai M.-J., Liao Y.-T., Lin Y.-S., Hu C.-J., Hsu Y.-H. (2022). Cognitive Function Associated with Gut Microbial Abundance in Sucrose and S-Adenosyl-L-Methionine (SAMe) Metabolic Pathways. J. Alzheimers Dis..

[114] Qian Y., Yang X., Xu S., Wu C., Song Y., Qin N., Chen S.-D., Xiao Q. (2018). Alteration of the Fecal Microbiota in Chinese Patients with Parkinson’s Disease. Brain Behav. Immun..

[115] Terzo S., Mulè F., Caldara G.F., Baldassano S., Puleio R., Vitale M., Cassata G., Ferrantelli V., Amato A. (2020). Pistachio Consumption Alleviates Inflammation and Improves Gut Microbiota Composition in Mice Fed a High-Fat Diet. Int. J. Mol. Sci..

[116] Wang M., Amakye W.K., Gong C., Ren Z., Yuan E., Ren J. (2022). Effect of Oral and Intraperitoneal Administration of Walnut-Derived Pentapeptide PW5 on Cognitive Impairments in APPSWE/PS1ΔE9 Mice. Free Radic. Biol. Med..

[117] Liang Y., Zhang Y., Deng Y., Liang S., He Y., Chen Y., Liu C., Lin C., Han L., Tu G. (2018). Chaihu-Shugan-San Decoction Modulates Intestinal Microbe Dysbiosis and Alleviates Chronic Metabolic Inflammation in NAFLD Rats via the NLRP3 Inflammasome Pathway. Evid. Based Complement. Alternat. Med..

[118] Monk J.M., Lepp D., Zhang C.P., Wu W., Zarepoor L., Lu J.T., Pauls K.P., Tsao R., Wood G.A., Robinson L.E. (2016). Diets Enriched with Cranberry Beans Alter the Microbiota and Mitigate Colitis Severity and Associated Inflammation. J. Nutr. Biochem..

[119] Kim S., Choi S., Dutta M., Asubonteng J.O., Polunas M., Goedken M., Gonzalez F.J., Cui J.Y., Gyamfi M.A. (2021). Pregnane X Receptor Exacerbates Nonalcoholic Fatty Liver Disease Accompanied by Obesity- and Inflammation-Prone Gut Microbiome Signature. Biochem. Pharmacol..

[120] O’Callaghan J., O’Toole P.W. (2013). Lactobacillus: Host-Microbe Relationships. Curr. Top Microbiol. Immunol..

[121] Pessione E. (2012). Lactic Acid Bacteria Contribution to Gut Microbiota Complexity: Lights and Shadows. Front. Cell Infect. Microbiol..

[122] Lee J., d’Aigle J., Atadja L., Quaicoe V., Honarpisheh P., Ganesh B.P., Hassan A., Graf J., Petrosino J., Putluri N. (2020). Gut Microbiota-Derived Short-Chain Fatty Acids Promote Poststroke Recovery in Aged Mice. Circ. Res..

[123] Emge J.R., Huynh K., Miller E.N., Kaur M., Reardon C., Barrett K.E., Gareau M.G. (2016). Modulation of the Microbiota-Gut-Brain Axis by Probiotics in a Murine Model of Inflammatory Bowel Disease. Am. J. Physiol. Gastrointest. Liver Physiol..

[124] Van Tongeren S.P., Slaets J.P.J., Harmsen H.J.M., Welling G.W. (2005). Fecal Microbiota Composition and Frailty. Appl. Environ. Microbiol..

[125] Aizawa E., Tsuji H., Asahara T., Takahashi T., Teraishi T., Yoshida S., Ota M., Koga N., Hattori K., Kunugi H. (2016). Possible Association of Bifidobacterium and Lactobacillus in the Gut Microbiota of Patients with Major Depressive Disorder. J. Affect. Disord..

[126] Wang Q.-J., Shen Y.-E., Wang X., Fu S., Zhang X., Zhang Y.-N., Wang R.-T. (2020). Concomitant Memantine and Lactobacillus Plantarum Treatment Attenuates Cognitive Impairments in APP/PS1 Mice. Aging.

[127] Lew L.-C., Hor Y.-Y., Yusoff N.A.A., Choi S.-B., Yusoff M.S.B., Roslan N.S., Ahmad A., Mohammad J.A.M., Abdullah M.F.I.L., Zakaria N. (2019). Probiotic Lactobacillus Plantarum P8 Alleviated Stress and Anxiety While Enhancing Memory and Cognition in Stressed Adults: A Randomised, Double-Blind, Placebo-Controlled Study. Clin. Nutr..

[128] Smith C.J., Emge J.R., Berzins K., Lung L., Khamishon R., Shah P., Rodrigues D.M., Sousa A.J., Reardon C., Sherman P.M. (2014). Probiotics Normalize the Gut-Brain-Microbiota Axis in Immunodeficient Mice. Am. J. Physiol. Gastrointest. Liver Physiol..

[129] Lee K., Kim J., Park S.-D., Shim J.-J., Lee J.-L. (2021). Lactobacillus Plantarum HY7715 Ameliorates Sarcopenia by Improving Skeletal Muscle Mass and Function in Aged Balb/c Mice. Int. J. Mol. Sci..

[130] Valcheva R., Hotte N., Gillevet P., Sikaroodi M., Thiessen A., Madsen K.L. (2015). Soluble Dextrin Fibers Alter the Intestinal Microbiota and Reduce Proinflammatory Cytokine Secretion in Male IL-10-Deficient Mice. J. Nutr..

[131] Long-Smith C., O’Riordan K.J., Clarke G., Stanton C., Dinan T.G., Cryan J.F. (2020). Microbiota-Gut-Brain Axis: New Therapeutic Opportunities. Annu. Rev. Pharmacol. Toxicol..

