# Supplementary material for: The Many Ages of Microbiome–Gut–Brain Axis
Source: Nutrients. 2022 Jul 18;14(14):2937. doi: 10.3390/nu14142937 (PMC9319041; doi:10.3390/nu14142937)
Supplement: Supplementary file 1 [file nutrients-14-02937-s001.zip › nutrients-1785490-supplementary.pdf]

nutrients

MDPI

Supplementary Materials

The Many Ages of Microbiome–Gut–Brain Axis

Daniela Ratto [1], Elisa Roda [2], Marcello Romeo [1], Maria Teresa Venuti [1], Anthea Desiderio [3], Giuseppe Lupo [3], Enrica Capelli [3], Anna Sandionigi [4,5] and Paola Rossi [1,*]

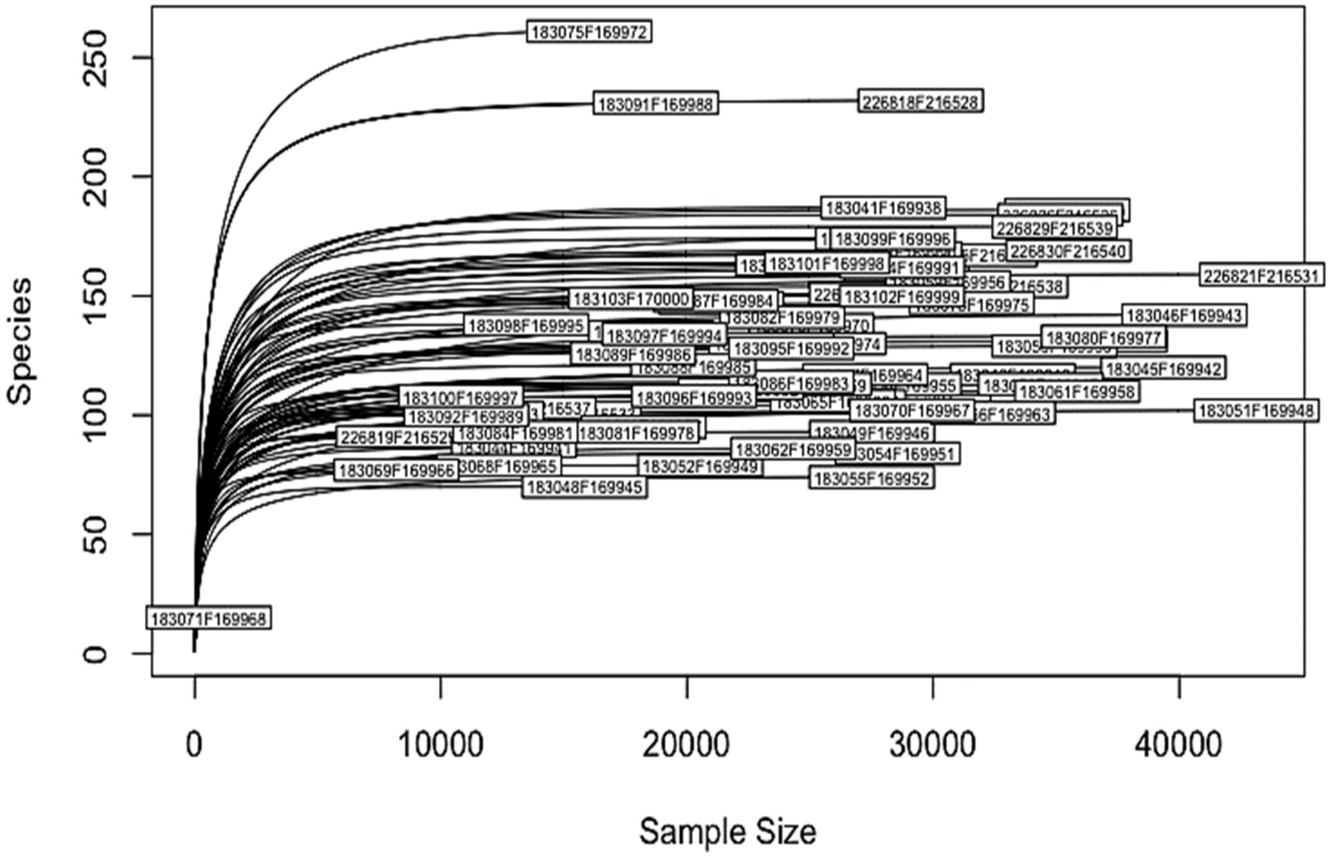

Figure S1. Rarefaction curve.

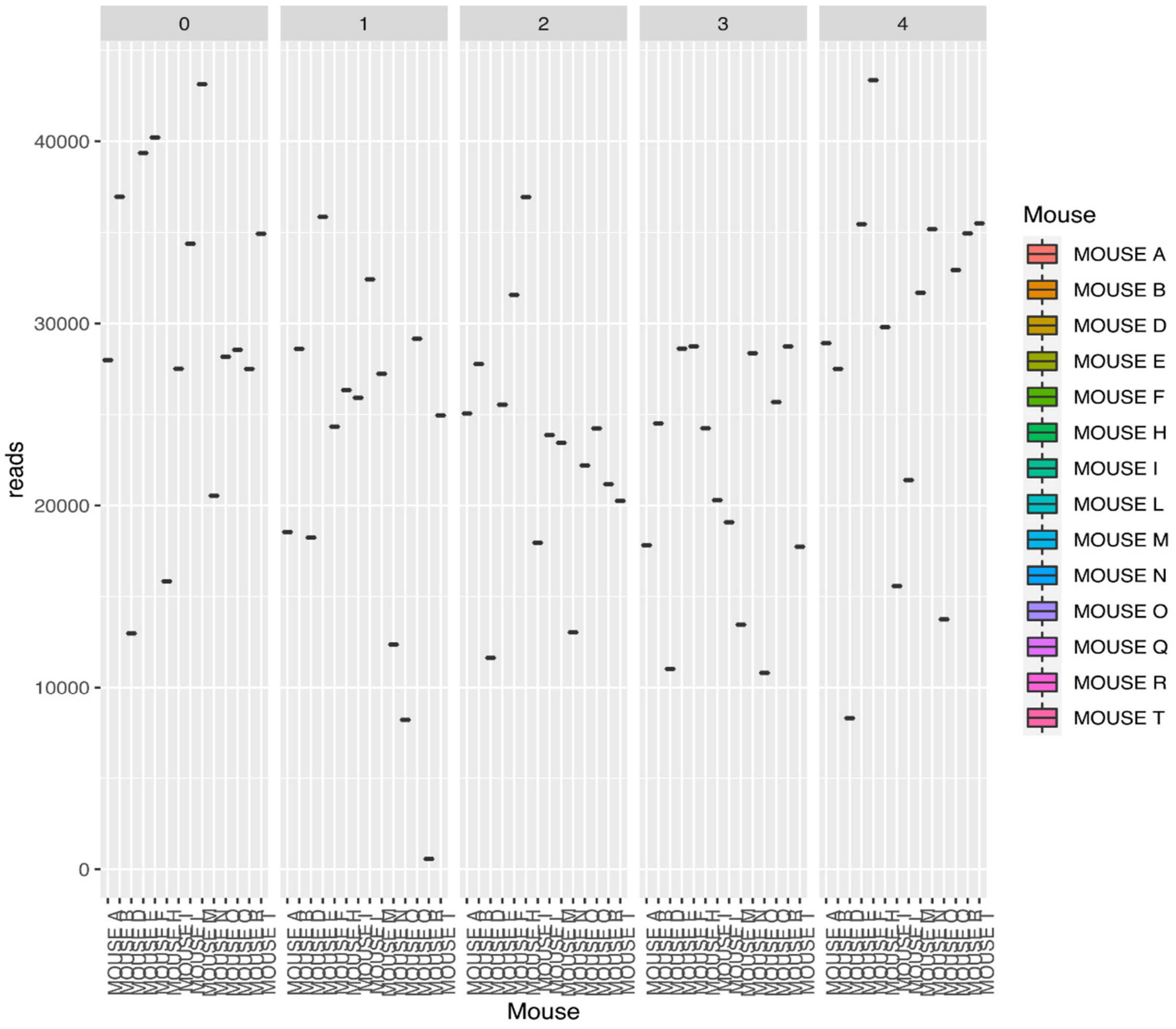

Figure S2. The graph shows the exact number of reads obtained per mice at each time.

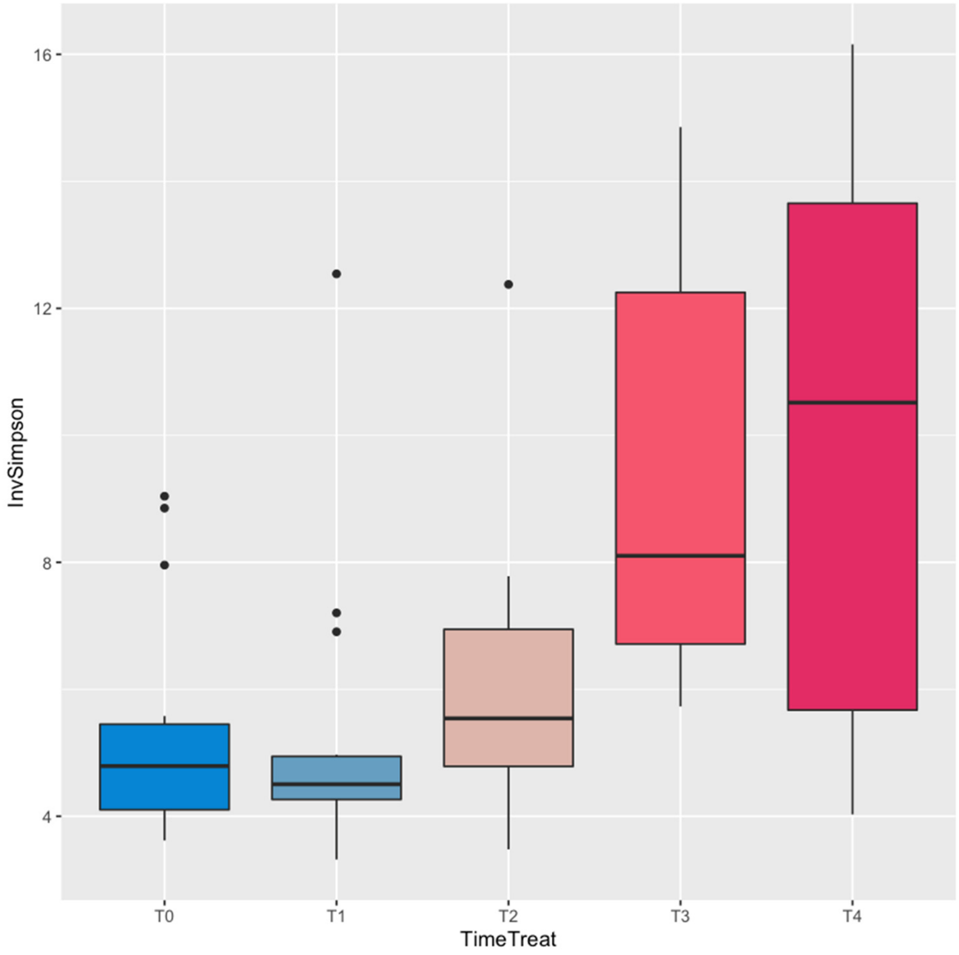

Figure S3. Plot of the Alpha diversity distribution by Shannon diversity index during aging (SDI estimated for each time point).

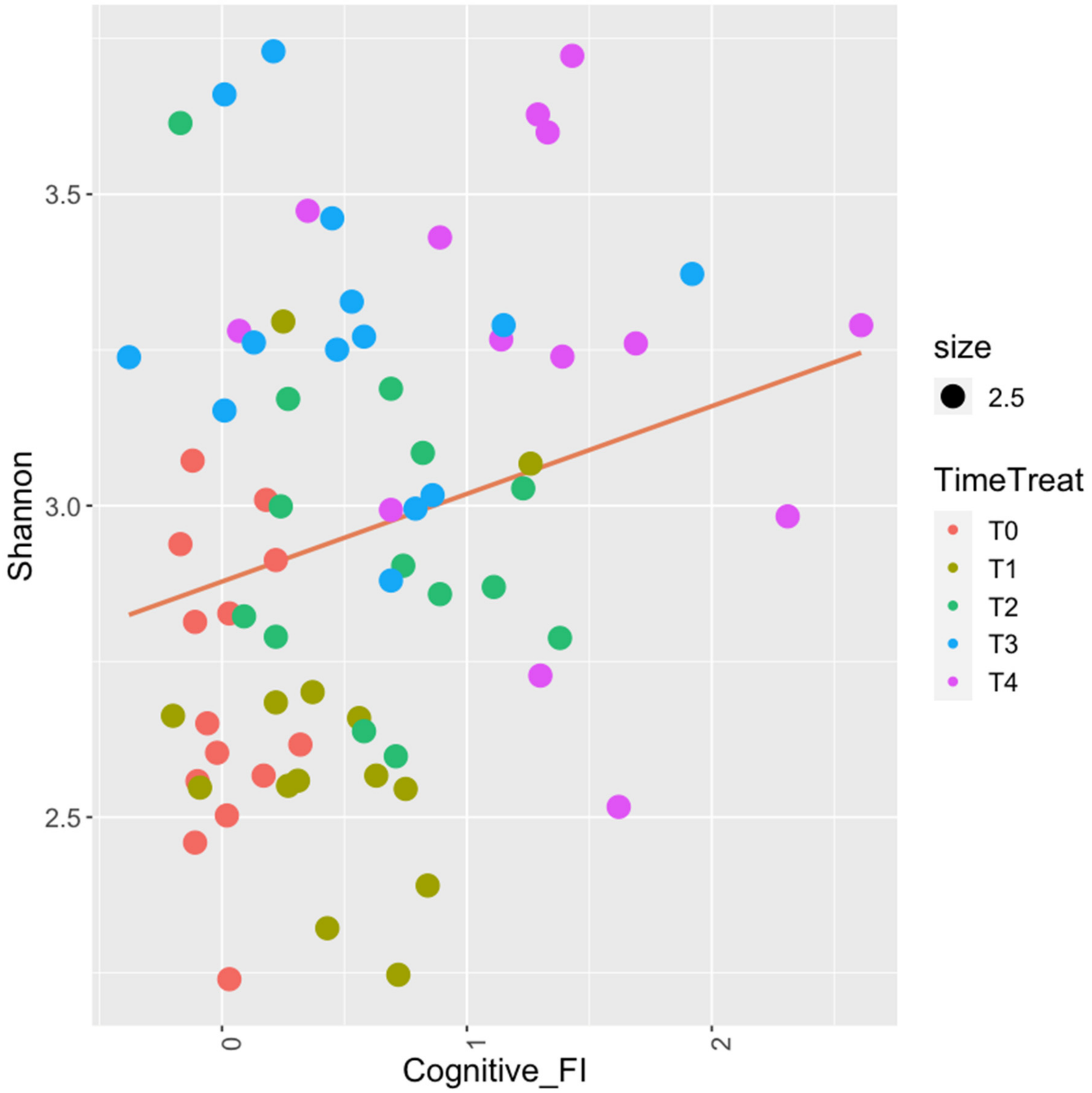

Figure S4. Figure showing the relationship between Shannon Index and Cognitive FI. Correlation between colors and experimental times as described in figure label.

Table S1. Statistical analysis of the effect of aging on alpha diversity based on Shannon diversity index (on the top) and Faith phylogenetic distance (on the bottom), including the comparison among all time points.

**Shannon Index**		
**Time vs. time**	**WSRT**	**FDR *P*-value**
T0 <-> T1	17	0.092
T0 <-> T2	9	0.004
T0 <-> T3	0	0.0001
T0 <-> T4	8	0.006
T1 <-> T2	6	0.007
T1 <-> T3	0	0.0005
T1 <-> T4	2	0.003
T2 <-> T3	13	0.011
T2 <-> T4	12	0.017
T3 <-> T4	41	0.787
**Faith PD Index**		
**Time vs. time**	**WSRT**	**FDR *P*-value**
T0 <-> T1	36	0.850
T0 <-> T2	47	0.761
T0 <-> T3	9	0.004
T0 <-> T4	0	0.0002
T1 <-> T2	31	0.569
T1 <-> T3	15	0.064
T1 <-> T4	0	0.001
T2 <-> T3	24	0.0785
T2 <-> T4	0	0.0002
T3 <-> T4	0	0.0002

Table S2. Statistical analysis of the effect of aging on beta diversity based on Bray-Curtis distance matrix, including the comparison among all time points.

Time vs. time	SumsOfSqs	MeanSqs	F.Model	R^2	*P*-value corrected
T0 <-> T1	0.1913481	0.1913481	2.200444	0.08398499	0.02997003
T0 <-> T2	0.4629483	0.4629483	4.264518	0.14090818	0.001427144
T0 <-> T3	0.9996884	0.9996884	7.221748	**0.21738013**	0.001427144
T0 <-> T4	1.4650306	1.4650306	9.073686	**0.266296**	0.001427144
T1 <-> T2	0.2045176	0.2045176	2.316692	0.08803127	0.003746254
T1 <-> T3	0.7464663	0.7464663	6.187632	0.20497242	0.001427144
T1 <-> T4	1.2260729	1.2260729	8.461537	**0.26894863**	0.001427144
T2 <-> T3	0.3513357	0.3513357	2.515884	0.08822747	0.001427144
T2 <-> T4	0.8314076	0.8314076	5.1092	0.16968901	0.001427144
T3 <-> T4	0.3013982	0.3013982	1.555269	0.05856725	0.108891109

Table S3. Summary results of LME used to estimate the effect of time on Cognitive FI.

	**Coef.**	**Std.Err.**	**z**	**P>|z|**	**[0.025**	**0.975]**
Intercept	0.101	0.108	0.935	0.35	−0.111	0.313
time	0.194	0.035	5.625	0	0.127	0.262
Group Var	0.014	0.046				

Table S4. Summary results of LME used to estimate the effect of time and Cognitive FI. on the Faith PD index (alpha diversity index).

**Cognitive LME**	**Coef.**	**Std.Err.**	**z**	**P>|z|**	**[0.025**	**0.975]**
Intercept	24.012	1.67	14.383	0	20.74	27.284
time	−1.331	0.756	−1.76	0.078	−2.814	0.151
Cognitive FI	6.345	3.252	1.951	0.051	−0.029	12.719
time:Cognitive FI	−2.994	0.961	−3.115	0.002	−4.879	−1.11
Group Var	10.271	1.325				

Table S5. Summary results of LME used to estimate the effect of time and Cognitive FI. on the beta diversity, based on Bray Curtis index.

	**Df**	**SumOfSqs**	**F**	**Pr(>F)**
Cognitive_FI	1	0.938	6.611	0.001
TimeTreat	4	1.977	3.483	0.001
Cognitive_FI:TimeTreat	4	0.446	0.786	0.852
Residual	57	8.088	NA	NA
